# CqHKT1 and CqSOS1 mediate genotype-dependent Na^+^ exclusion under high salinity conditions in quinoa

**DOI:** 10.3389/fpls.2025.1597647

**Published:** 2025-06-18

**Authors:** Yasufumi Kobayashi, Ryohei Sugita, Miki Fujita, Yasuo Yasui, Yoshinori Murata, Takuya Ogata, Yukari Nagatoshi, Yasunari Fujita

**Affiliations:** ^1^ Biological Resources and Post-harvest Division, Japan International Research Center for Agricultural Sciences (JIRCAS), Tsukuba, Ibaraki, Japan; ^2^ Radioisotope Research Center, Nagoya University, Nagoya, Aichi, Japan; ^3^ Mass Spectrometry and Microscopy Unit, RIKEN Center for Sustainable Resource Science, Tsukuba, Ibaraki, Japan; ^4^ Graduate School of Agriculture, Kyoto University, Kyoto, Japan; ^5^ Food Program, Japan International Research Center for Agricultural Sciences (JIRCAS), Tsukuba, Ibaraki, Japan; ^6^ Graduate School of Life Environmental Science, University of Tsukuba, Tsukuba, Ibaraki, Japan

**Keywords:** quinoa, salt stress, CqHKT1, CqSOS1, inbred lines, genotype

## Abstract

Salinity threatens crop production worldwide, and salinized areas are steadily increasing. As most crops are sensitive to salt, there is a need to improve the salt tolerance of major crops and promote the cultivation of under-utilized salt-tolerant crops. Quinoa, a pseudocereal and leafy vegetable from the Andean region of South America, is highly salt-tolerant, thrives in marginal environments, and has excellent nutritional properties. Research has often focused on epidermal bladder cells, a feature of quinoa thought to contribute to salt tolerance; however, recent evidence suggests that these cells are not directly involved. The salt tolerance mechanism in quinoa remains unclear. Here, we show genotype-dependent differences in Na^+^ and K^+^ accumulation mechanisms using representative 18 lines of three genotypes by focusing on young quinoa seedlings at a stage without epidermal bladder cells. High salinity (600 mM NaCl) did not affect the early growth of all three quinoa genotypes. Under high salinity conditions, lowland quinoa lines tended to accumulate more Na^+^ in their aerial parts than highland lines did. By contrast, K^+^ accumulation was slightly reduced in the aerial parts but significantly decreased in the roots of all the genotypes. Resequencing of 18 quinoa lines supports the notion that genotype determines aboveground Na^+^ uptake and gene expression in response to high salinity. Using virus-induced gene silencing, we further demonstrated that CqHKT1 and CqSOS1 mediate Na^+^ exclusion in quinoa. These findings provide insight into salt tolerance mechanisms, serving as a basis for improving crop production under high salinity conditions.

## 1 Introduction

Salinity limits crop production in many regions of the world, and the affected areas and resulting economic losses are expected to increase (Melino and Tester, 2023). Recently, not only has the FAO’s Global Map of Salt Affected Soils (GSASmap) covering 118 countries been released (FAO, 2021), but it has also been reported that there are 17 million square kilometers of salt-affected soils on the planet (Negacz et al., 2022). However, many crops, including staples such as maize (*Zea mays*), wheat (*Triticum aestivum*), rice (*Oryza sativa*), and soybean (*Glycine max*), are non-salt-tolerant plants. Salt-tolerant plants are relatively rare, comprising less than 0.25% of flowering plants (Bromham, 2015). Therefore, there is a growing need to confer salt tolerance to conventional crops and to take advantage of salt-tolerant crops such as quinoa (*Chenopodium quinoa* Willd.). Deciphering the salt tolerance mechanisms of salt-tolerant plants may provide insights for improving salt tolerance in non-salt-tolerant crops (Melino and Tester, 2023).

Quinoa is an annual C3 pseudocereal and leafy vegetable of the Amaranthaceae family, which also includes sugar beet (*Beta vulgaris* L.) and spinach (*Spinacia oleracea* L.). Quinoa is emerging as a potentially critical crop for global food and nutrition security due to its excellent nutritional value (Navruz-Varli and Sanlier, 2016; Kobayashi et al., 2024) and ability to thrive in marginal environments (Bonifacio, 2019). Quinoa grows at a wide range of altitudes, from coastal areas to around 4,000 meters above sea level (Jacobsen, 2003; Zurita-Silva et al., 2014; Bonifacio, 2019). Quinoa can tolerate drought, high salinity, and frost, as exemplified by its cultivation near the Salar de Uyuni in Bolivia (Jacobsen, 2003; Hariadi et al., 2011; Zurita-Silva et al., 2014; Yasui et al., 2016; Bonifacio, 2019; Mizuno et al., 2020).

Plants have evolved not only morphological plasticity but also various physiological mechanisms, such as ion accumulation and exclusion, osmotic regulation, enhanced antioxidant responses, and ion homeostasis, that allow them to maintain growth under high salinity conditions (Munns and Tester, 2008; Adolf et al., 2013; Deinlein et al., 2014; van Zelm et al., 2020). In terms of morphological characteristics, extensive research has explored how epidermal bladder cells on the leaf surface of salt-tolerant plants such as quinoa and common ice plants (*Mesembryanthemum crystallinum* L.), both of which are in the Caryophyllales order, contribute to salt stress tolerance (Agarie et al., 2007; Flowers and Colmer, 2008; Yuan et al., 2016; Kiani-Pouya et al., 2017). Epidermal bladder cells on the surface of aerial parts are one of the most striking morphological features, and their role in salt stress tolerance has been extensively studied (Adolf et al., 2013; Kiani-Pouya et al., 2017; Böhm et al., 2018; Kiani-Pouya et al., 2019; Imamura et al., 2020; Bazihizina et al., 2022; Moog et al., 2023). Recent studies have shown that the salt tolerance of quinoa mutants lacking epidermal bladder cells is similar to that of the wild type and that K^+^ accumulates preferentially in quinoa epidermal bladder cells over Na^+^, indicating that epidermal bladder cells are directly involved in the salt tolerance mechanism (Moog et al., 2022). More recently, epidermal bladder cells in quinoa were implicated in herbivore defense mechanisms (Moog et al., 2023). These findings compel us to look beyond morphological features such as epidermal bladder cells in efforts to decipher the salt tolerance mechanism in quinoa. While abscisic acid (ABA)–mediated osmotic regulation (Fujita et al., 2011) and enhancement of antioxidant responses (Choudhury et al., 2017) are common responses to abiotic stresses such as drought and salinity (Zhu, 2002; Golldack et al., 2014; Gupta et al., 2020), the mechanisms specifically required to respond for the plant’s response to salt stress are ion transport and ion homeostasis (Munns and Tester, 2008; Deinlein et al., 2014; van Zelm et al., 2020). Molecular genetics and plant electrophysiological studies have shown that the ability to maintain high cytosolic K^+^/Na^+^ ratios is important for salt tolerance in plants to maintain ion homeostasis (Shabala and Cuin, 2008). Transporters such as HKT1 and SOS1 are required for Na^+^ efflux from plants (Munns and Tester, 2008; Deinlein et al., 2014; van Zelm et al., 2020) and are considered to be among the most important transporters involved in salt stress tolerance in a wide range of plant species (Yang and Guo, 2018; Ali et al., 2021). In the model plant Arabidopsis (*Arabidopsis thaliana*), the plasma membrane Na^+^/H^+^ antiporter SOS1 mediates root Na^+^ efflux and xylem Na^+^ loading, and the channel-like protein HKT1;1 functions in xylem Na^+^ unloading to regulate net Na^+^ uptake (Deinlein et al., 2014; van Zelm et al., 2020). A recent study showed that the Ca^2+^ sensor protein SOS3 inversely regulates the two major Na^+^ transporters SOS1 and HKT1 to mediate Na^+^ loading and unloading at the xylem in Arabidopsis plants, respectively (Gámez-Arjona et al., 2024). In quinoa, it remains to be determined whether transporters such as SOS1 and HKT1 play a role in salt tolerance *in planta*, as observed in model plant studies.

Quinoa is an allotetraploid with 2*n* = 4*x* = 36 chromosomes, comprising two subgenomes, A and B (Ward, 2000; Palomino et al., 2008; Yangquanwei et al., 2013; Kolano et al., 2016; Heitkam et al., 2020). The complexity of the genome due to allotetraploidy and the genetic heterogeneity due to partial outcrossing resulting from the presence of both hermaphroditic and female flowers on the same plant have hampered molecular analysis of quinoa (Maughan et al., 2004; Christensen et al., 2007). Our group (Yasui et al., 2016) and three subsequent independent groups (Jarvis et al., 2017; Zou et al., 2017; Bodrug-Schepers et al., 2021) have reported the draft genome sequences of quinoa. We generated approximately 140 genotyped quinoa inbred lines suitable for molecular analysis, including Kd, a genome-sequenced and standard quinoa inbred line, and demonstrated genotype–phenotype relationships among the inbred lines for salt tolerance and key growth traits (Mizuno et al., 2020). We also showed that the quinoa inbred lines can be divided into three genetic subpopulations: the northern highland, southern highland, and lowland (Mizuno et al., 2020). Recently, high-quality chromosome-level genome assemblies have been reported by our groups for the northern and southern highland lines, J075 and J100, respectively (Kobayashi et al., 2024), and by another independent group for the lowland line QQ74 (Rey et al., 2023). Furthermore, we have developed a technique to analyze the function of endogenous genes in quinoa using virus-induced gene silencing (VIGS) and virus-mediated overexpression, thereby advancing the field of functional genomics analysis (Ogata et al., 2021).

In the present study, we focused on young quinoa seedlings lacking epidermal bladder cells and examined the physiological responses of all three genotypic lines such as lowland lines (J028, J045, J079, J082, J122, and Kd), northern highland lines (J064, J071, J072, J073, J074, and J075), and southern highland lines (J054, J094, J096, J099, J100, and J128) to high salinity (600 mM NaCl). To further explore the mechanism of salt tolerance in quinoa, we also performed transcriptome analysis, genomic analysis using 18 resequencing data, and VIGS analysis of transporter genes in quinoa plants. We show that CqHKT1 and CqSOS1 are involved in the genotype-dependent Na^+^ exclusion in quinoa, providing insight into salt tolerance mechanisms in quinoa.

## 2 Materials and methods

### 2.1 Plant materials, growth conditions, and salt treatments

The quinoa inbred lines (Yasui et al., 2016; Mizuno et al., 2020; Ogata et al., 2021) used in this study are listed in **Supplementary Table 1**. For salt stress tests, quinoa seeds were sown and grown in vermiculite-filled cell trays in 8.2-L trays containing reverse osmosis (RO) water for 10 days in a growth chamber set at 22 ± 2°C under a 12-h-light/12-h-dark photoperiod, with light supplied at a 75 ± 25 µmol photons/m^2^/s. Ten days after sowing, these cell trays were transferred to trays containing 0 or 600 mM NaCl RO water. After the salt or control treatment, individual plants were pulled from their cells at the designated time and cut into roots, hypocotyls, and cotyledons for sampling. For VIGS analysis, quinoa Kd seeds were sown in a peat moss mixture (Jiffy Mix, Sakata Seeds, Yokohama, Japan) in a cell tray. After 7 days, the seedlings were transferred to a standard potting mix (Tsuchitaro, Sumitomo Forestry, Tokyo, Japan) in 0.11-L pots and grown in a temperature-controlled phytotron set at 22 ± 2°C under a 12-h-light/12-h-dark photoperiod for 7 days.

### 2.2 Determination of Na^+^ and K^+^ ions in quinoa seedling tissues

The Na^+^ and K^+^ measurements were performed as previously described (Cataldi et al., 2003), with slight modifications. Cotyledon, hypocotyl, and root tissues of quinoa seedlings were collected after salt treatment and oven-dried at 60°C for 2 days. Tissue extracts were prepared by adding 1 mL ultrapure water to ground tissue using a ShakeMaster (BMS, Japan) and 2-mm zirconia beads (1,100 rpm, 2min). The suspensions were heated at 90°C for 20 min and centrifuged at 21,500 *g* for 15 min at 4°C. The ionic contents were analyzed using a high-performance liquid chromatography (HPLC) system (Jasco, Japan, PU-4185 pump, CO-4060 column oven, AS-4150 autosampler) with a CD-200 conductivity detector (Shodex, Japan). Na^+^ and K^+^ were separated using a Shim-pack IC-C4 with Shim-pack IC-C4 guard column (Shimadzu, Japan) and eluted with 2.5 mM oxalic acid. The peak area and concentration of the ions were calculated using ChromNAV Ver.2 software (Jasco, Japan).

### 2.3 ^22^Na experiment for sodium uptake assay

Quinoa Kd, J075, and J100 seeds were sown on mesh floating in a modified Molecular Genetics Research Laboratory (MGRL) solution (Kobayashi et al., 2013) containing 0.175 mM sodium phosphate buffer, 0.4 mM NaNO_3_, 0.3 mM KNO_3_, 0.2 mM CaCl_2_, 0.15 mM MgSO_4_, 3 μM H_3_BO_3_, 0.86 μM Fe(III)-EDTA, 1.03 μM MnSO_4_, 0.1 μM CuSO_4_, 0.1 μM ZnSO_4_, 0.24 nM (NH_4_)_6_Mo_7_O_24_ 4H_2_O, 1.3 nM CoCl_2_, and 2 mM MES (pH 5.6). After 6 days, the seedlings were transferred to the MGRL solution containing 21.7 pM ^22^Na (1 kBq/mL, Chiyoda Technol Co. Japan). The seedlings were collected at 1 and 24 h after the onset of ^22^Na treatment. After the seedling roots were rinsed with the modified MGRL solution, ^22^Na activity was measured using imaging plates (BAS-IP MS, FUJIFILM, Tokyo, Japan) and a Typhoon FLA-7000 image reader (Cytiva, Tokyo, Japan). The ^22^Na content of the aerial parts of each quinoa line was calculated from the photostimulated luminescence value using a calibration curve generated from spotted samples.

### 2.4 Gene expression analysis

Total RNA extraction and reverse transcription quantitative PCR (RT-qPCR) analysis were generally performed as previously described (Nagatoshi et al., 2023). Cotyledons, hypocotyls, and roots of seedlings treated as described for Na^+^ and K^+^ ion content measurements were rapidly frozen in liquid nitrogen. RNA was extracted from tissues using RNAiso plus (Takara). First-strand cDNA was synthesized from half a volume of total RNA (1 μg) treated for 30 min at 37°C with RQ1 RNase-free DNase (Promega) using a SuperScript III First-Strand Synthesis System (Invitrogen). Gene expression was quantified on a QuantStudio 7 Frex real-time PCR system (Applied Biosystems) using GoTaq qPCR Master Mix (Promega). The primers used in this study are described in **Supplementary Table 2**.

### 2.5 RNA-seq analysis

Three quinoa inbred lines (Kd, J075, and J100) grown in vermiculite culture for 10 d were treated with 0 or 600 mM NaCl solution for 0, 6, and 24 h. Total RNA was extracted from cotyledons and roots and treated with DNase as described above. The libraries were prepared and sequenced at Macrogen Japan. The integrity of the total RNA was evaluated using an Agilent 2100 Bioanalyzer (Agilent). Paired-end sequencing (151-bp) of the mRNA library prepared using a TruSeq standard mRNA library Prep was performed on a NovaSeq 6000. All paired-end reads were trimmed using a Trimmomatic (v.0.38) (Bolger et al., 2014) to remove adapter sequence (ILLUMINACLIP:2:20:10) and low-quality reads (SLIDINGWINDOW:4:20, MINLEN:40). The filter-passed reads were mapped on reference sequence from CoGe database (id60716) (Rey et al., 2023) using Hisat2 (v2.1.0) (Kim et al., 2019). Reads mapped to transcript regions were counted and transcripts per million (TPM) values of transcripts were calculated for each sample.

### 2.6 Differential gene expression analysis

Differentially expressed genes (DEGs) were identified by organ-specific comparison of cotyledons and roots for 6 and 24 h of salt treatment. We performed quantitative analyses using three biological replicates. The statistical difference of read count data between the 0 and 600 mM NaCl treatments in each line was calculated using the edgeR package (Chen et al., 2016). Gene expression was considered significant if the false discovery rate (FDR) was less than 0.01 and |log_2_(fold change (FC))| was greater than 1 in at least one comparison. The resulting gene expression level data obtained were then imported into R software for hierarchical clustering. Prior to the clustering analysis, the TPM values were converted to z-scores for each gene using the “genescale” function included in the “genefilter” package obtained from bioconductor.org. Hierarchical clustering analysis and drawing of heat maps with dendrograms were performed using the heatmap.2 function of the “gplots” package (Warnes et al., 2024).

### 2.7 Functional enrichment analysis of DEGs in each cluster

Gene ontology (GO) enrichment analysis was performed with topGO using the classical algorithm with Fisher’s test (Alexa and Rahnenführer, 2009). GO annotations were also assigned to the quinoa reference genomes using the Trinotate pipeline (Bryant et al., 2017). The top 50 biological process GO terms significantly enriched in the gene list for each cluster were filtered by applying a 0.05 cutoff to Fisher’s weighted *P*-values.

### 2.8 AlphaFold predictions

The three-dimensional (3D) structures of the HKT1 and SOS1 transporter proteins were determined using AlphaFold 2 on Google Colab’s Notebook (Mirdita et al., 2022). Predictions were performed using the default LocalColabFold settings with 3 prediction cycles and no template mode. Output PDB files, predicted local difference distance (pLDDT) confidence scores, and predicted alignment error (PAE) were visually inspected individually for each transporter prediction. For each transporter, AlphaFold generated five atomic models, ranked in order of predicted accuracy. For simplicity, only the top-ranked model is shown in this manuscript.

### 2.9 Whole-genome resequencing

We used total genomic DNA from 17 quinoa inbred lines for whole-genome resequencing (Mizuno et al., 2020). DNA libraries were prepared using an Illumina TruSeq DNA PCR-Free Kit and sequenced as 151-bp paired-end reads on an Illumina NovaSeq 6000 system at Macrogen Japan. Low-quality reads and adapters were trimmed using Trimmomatic (v.0.38) (Bolger et al., 2014) with the options ‘SLIDINGWINDOW:4:25’ and ‘MINLEN:40’. The trimmed paired-end reads were aligned to the quinoa reference genome (QQ74, v2, id60716) from CoGe (https://genomevolution.org/coge/) using BWA 0.7.17 (Li, 2013). Samtools (v1.8) (Danecek et al., 2021) was used to convert the alignment SAM files to BAM files and prepare the alignment file for viewing in the Integrative Genomics Viewer (IGV) (Robinson et al., 2011).

### 2.10 Molecular cloning and plasmid construction

Based on the coding sequences (CDSs) for putative *CqHKT1;1, CqHKT1;2* and *CqSOS1* genes obtained from the NCBI database of *Chenopodium quinoa*, which the amino acid sequences of *Arabidopsis thaliana* AtHKT1 (AT4G10310) and AtSOS1 (AT2G01980) were used as queries in the BLASTP program, we designed PCR primers (**Supplementary Table 2**) to amplify trigger regions for VIGS in the CDSs. Apple latent spherical virus (ALSV)-RNA2 vectors for VIGS analyses were constructed as previously described (Ogata et al., 2021), with minor modifications. The amplified DNA fragments containing trigger sequences of quinoa genes for VIGS were cloned in-frame into the *Xho*I/*Bam*HI site of pEALSR2 to generate pEALSR2-CqHKT1;1, pEALSR2-CqHKT1;2 and pEALSR2-CqSOS1, respectively.

### 2.11 Virus inoculation and salt treatment

ALSV inoculations were performed as previously described (Ogata et al., 2021). The plasmids for the ALSV-RNA1 (pEALSR1) and ALSV-RNA2 constructs were mixed in equal amounts, and the DNA solution was mechanically inoculated onto the true leaves of 14-day-old quinoa plants (Iw inbred line) using carborundum (Li et al., 2004). The inoculated quinoa plants were grown for 2 to 3 weeks and the uninoculated upper leaves showing chlorotic spots symptoms were harvested. The detached leaves were ground using the ShakeMaster and 3-mm stainless steel beads and suspended in extraction buffer (0.1 M Tris-HCl, pH 8.0, 0.1 M NaCl, 5 mM MgCl_2_) (Igarashi et al., 2009). Debris was precipitated by centrifugation at 18,800 *g* for 10 min at 4°C, and the supernatants were used to inoculate quinoa Kd lines grown in the temperature-controlled phytotron as described above. Infected leaves or the inocula were stored at −80°C prior to use. ALSV derived from quinoa Iw plants inoculated with a mixture of pEALSR1 and pEALSR2 was used as a control (ALSV-WT). Seedlings (14-day-old) of quinoa Kd lines were mechanically inoculated with the inocula using carborundum. Kd plants at 10 days post inoculation were treated with 0.5 L of 300 mM NaCl solution once a week for 3 weeks. For Na^+^ content measurements, the lower leaves above the ALSV-inoculated leaves were collected and oven-dried at 60°C for 2 days. In the same plants, other leaves at similar positions were collected for gene expression analysis. Na^+^ content measurement and gene expression analysis were performed as described above.

### 2.12 Statistical analysis

All data are presented as mean ± standard deviation. Significant differences in data for physiological and morphological indices were determined by one-way analysis of variance and Tukey’s honestly significant difference (HSD) test or Student’s *t*-test using R version 4.3.0. The data were visualized using the “ggplot2” packages in R.

## 3 Results

### 3.1 Quinoa model line Kd thrives in 600 mM NaCl and accumulates Na^+^ in the aerial parts

**Figure 1:**
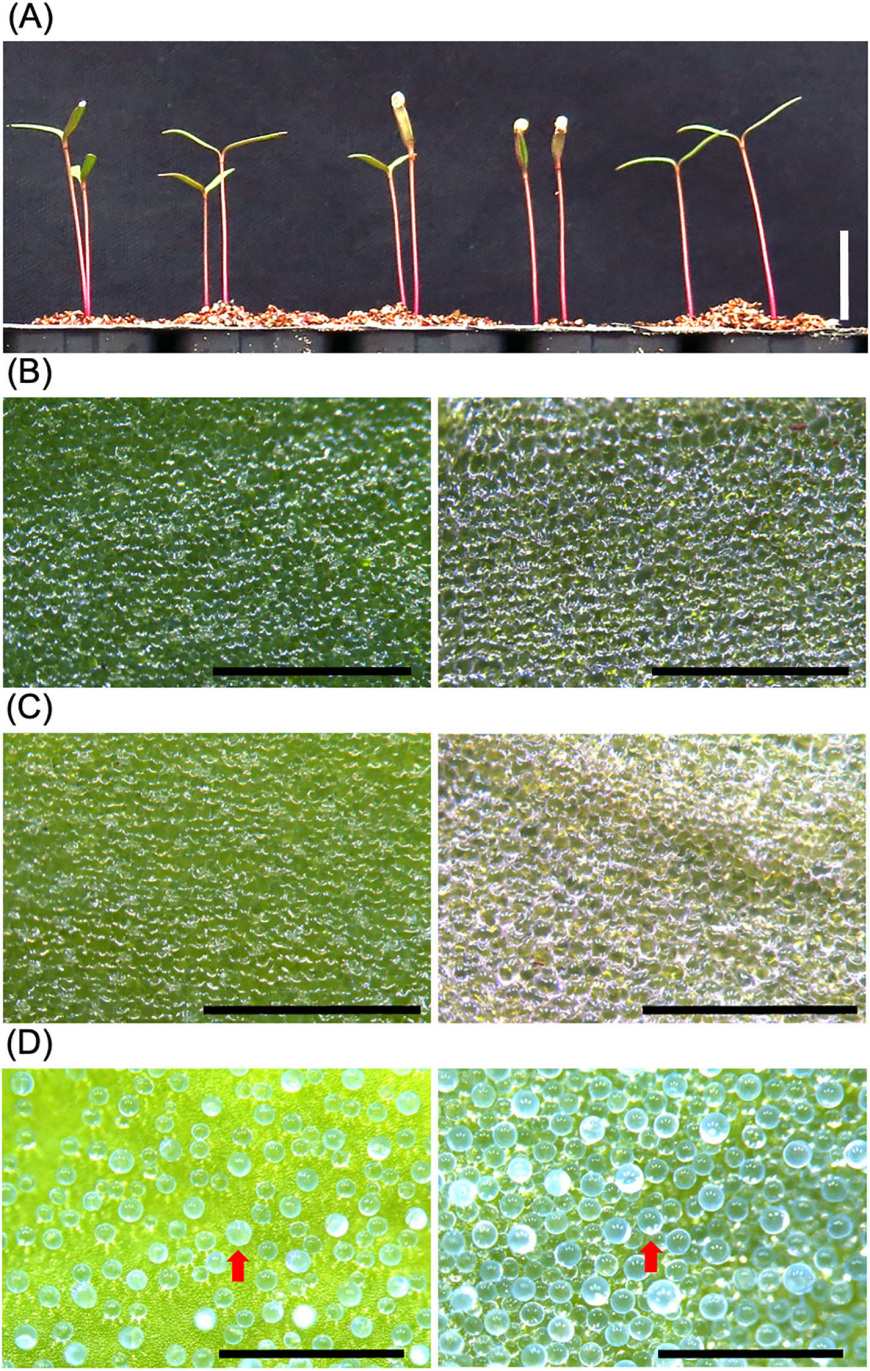
The cotyledons of young quinoa seedlings lack epidermal bladder cells. **(A)** Young seedlings of the Kd line, a representative model line of quinoa, were grown on vermiculite with RO water for 10 days. Bar, 1 cm. **(B–D)** Epidermal surfaces of cotyledons **(B, C)** and developing true leaf **(D)** of Kd plants were observed by a stereomicroscope. The Kd plants grown on standard potting mix for 3 weeks **(D)** were treated with 0 mM **(B)** or 600 mM **(C)** NaCl for 1 week. Left and right panels indicate adaxial and abaxial surfaces, respectively. Red arrows indicate representative epidermal bladder cells. Bars, 1 mm.

**Figure 2:**
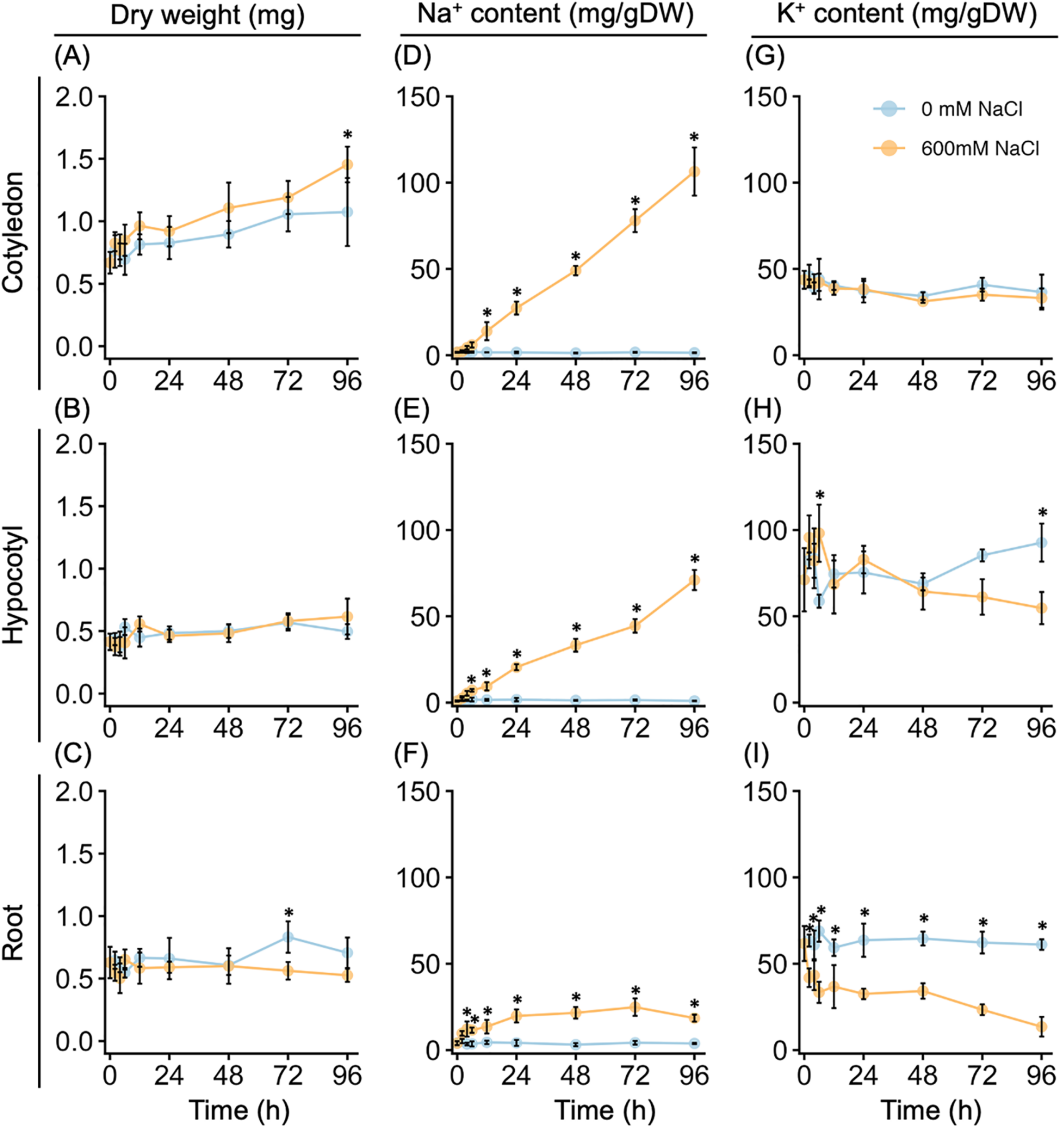
Growth and Na^+^ and K^+^ contents of young quinoa seedlings in response to high salinity over time. Ten-day-old quinoa seedlings grown on a vermiculite culture system were treated with 0 mM or 600 mM NaCl for 96 h. Cotyledon (top), hypocotyl (middle), and root (bottom) tissues were collected at 0, 2, 4, 6, 12, 24, 48, 72, and 96 h after the salt or control treatment. Dry weight **(A–C)** and Na^+^ **(D–F)** and K^+^ **(G–I)** contents were measured in each tissue per seedling. Error bars indicate SD (*n* = 5). *P < 0.05, one-way analysis of variance with Tukey’s HSD test was used to evaluate differences between seedlings subjected to the 600 mM NaCl treatment versus the 0 mM NaCl treatment at each time point.

In this analysis, we used Kd, a sequenced model quinoa line that exhibits uniform growth among individuals and is suitable for molecular biology experiments (Yasui et al., 2016; Mizuno et al., 2020; Ogata et al., 2021) (**Figures 1**, **2**). Although there is research supporting the notion that epidermal bladder cells are not involved in quinoa salt tolerance (Moog et al., 2022), to further confirm this notion, this study focused on the salt tolerance mechanism of quinoa plants at the seedling stage, which lack epidermal bladder cells (**Figure 1A**). In the quinoa plants examined in this study, no epidermal bladder cells were present on the cotyledons (**Figures 1B, C**), but epidermal bladder cells form on the first true leaves and all subsequent leaves that emerge (**Figure 1D**). In addition, in our previous research (Mizuno et al., 2020), the germination of quinoa in 600 mM NaCl showed clear differences depending on the genotype, in contrast to the case of 300 mM NaCl, so in this study we used 600 mM NaCl for the salt treatment.

We determined the dry weight and Na^+^ and K^+^ accumulations of various tissues of quinoa seedlings up to 4 days after treatment with or without 600 mM NaCl. Notably, salt treatment did not appear to inhibit biomass increase at most time points up to after 96 h of the salt treatment (**Figures 2A–C**). For all plants examined, the dry weight of the cotyledons increased in a time-dependent manner, whereas the dry weight of the hypocotyls and roots remained mostly unchanged (**Figures 2A–C**). In the salt-treated plants, the cotyledons and hypocotyls showed a time-dependent increase in Na^+^ accumulation, whereas there was no increase in Na^+^ accumulation in control plants (**Figures 2D, E**). Additionally, the Na^+^ accumulation in the roots plateaued after 24 h of salt treatment, all but the earliest time points were higher than in the control plants (**Figure 2F**). No difference in K^+^ accumulation was observed between the cotyledons of the salt-treated and control plants (**Figure 2G**); however, the K^+^ accumulation was lower in the roots of the salt-treated plants than in those of the control plants after 2 h of salt treatment (**Figure 2I**). By contrast, K^+^ accumulation in the hypocotyls increased in salt-treated plants but decreased in control plants up to after 6 h of salt treatment (**Figure 2H**). After 12 to 48 h of the salt treatment, there was no difference in K^+^ accumulation between the salt-treated and control plants, but after this time point, K^+^ increased in the control seedlings and decreased in the salt-treated plants (**Figure 2H**). Thus, the biomass increase of the model quinoa line Kd was not affected by treatment with 600 mM NaCl, but high salinity resulted in the accumulation of Na^+^, mainly in the aerial parts of the plants.

### 3.2 High salinity does not affect the early seedling growth of all three quinoa genotypes

**Figure 3:**
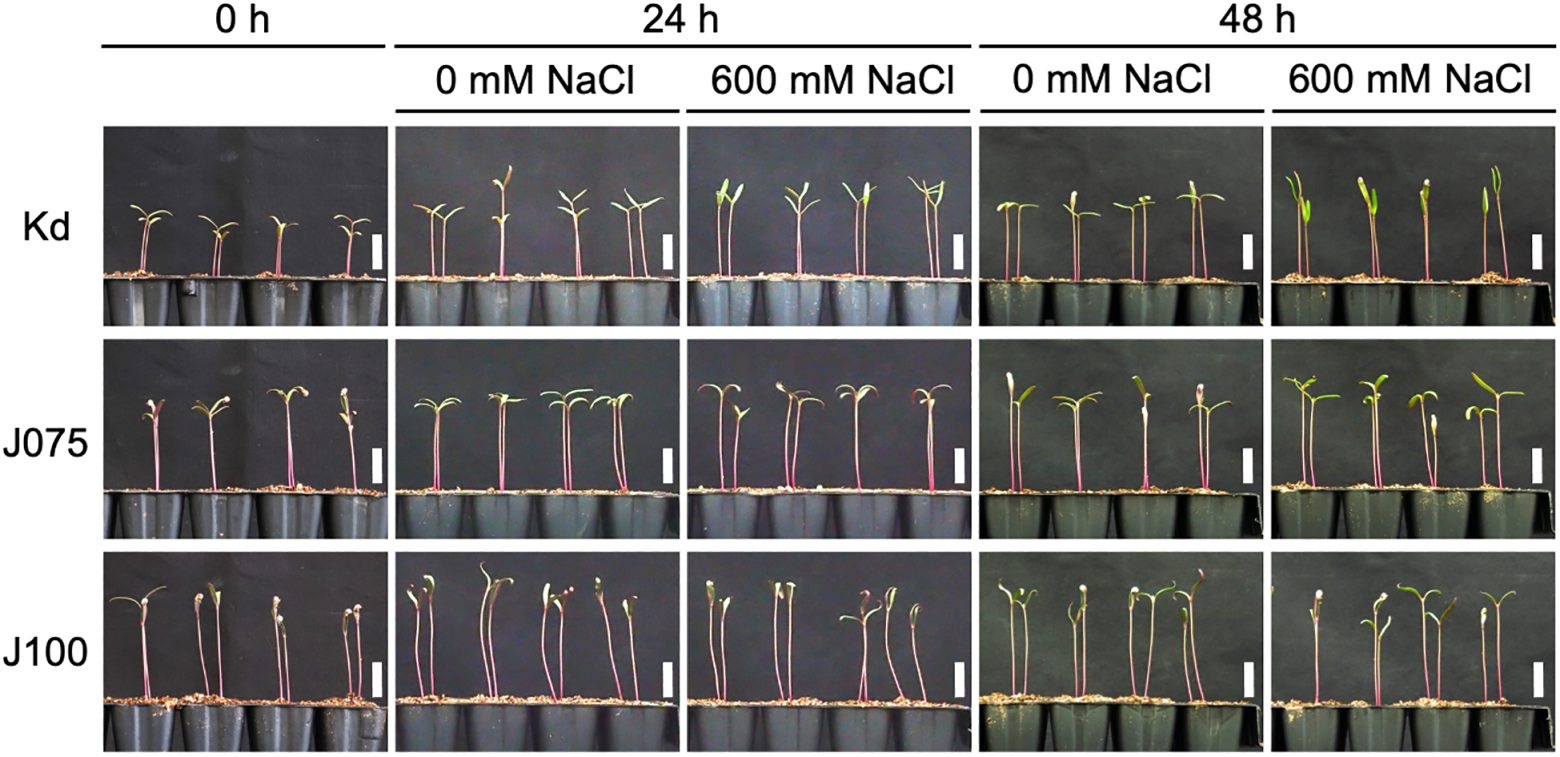
Growth of representative quinoa inbred lines of each genotype during salt stress test. Ten-day-old seedlings of quinoa inbred lines were treated with 0 mM or 600 mM NaCl for 0, 24 and 48 h. Photographs showing representative growth of Kd, a representative inbred line of lowland quinoa, J075, a representative inbred line of northern highland quinoa, and J100, a representative inbred line of southern highland quinoa. Lowland quinoa inbred line Kd, treated with 600 mM NaCl for 24 and 48 h, exhibited rapid upward (hyponastic) leaf movement not seen in the other lines. Bars, 1 cm.

**Figure 4:**
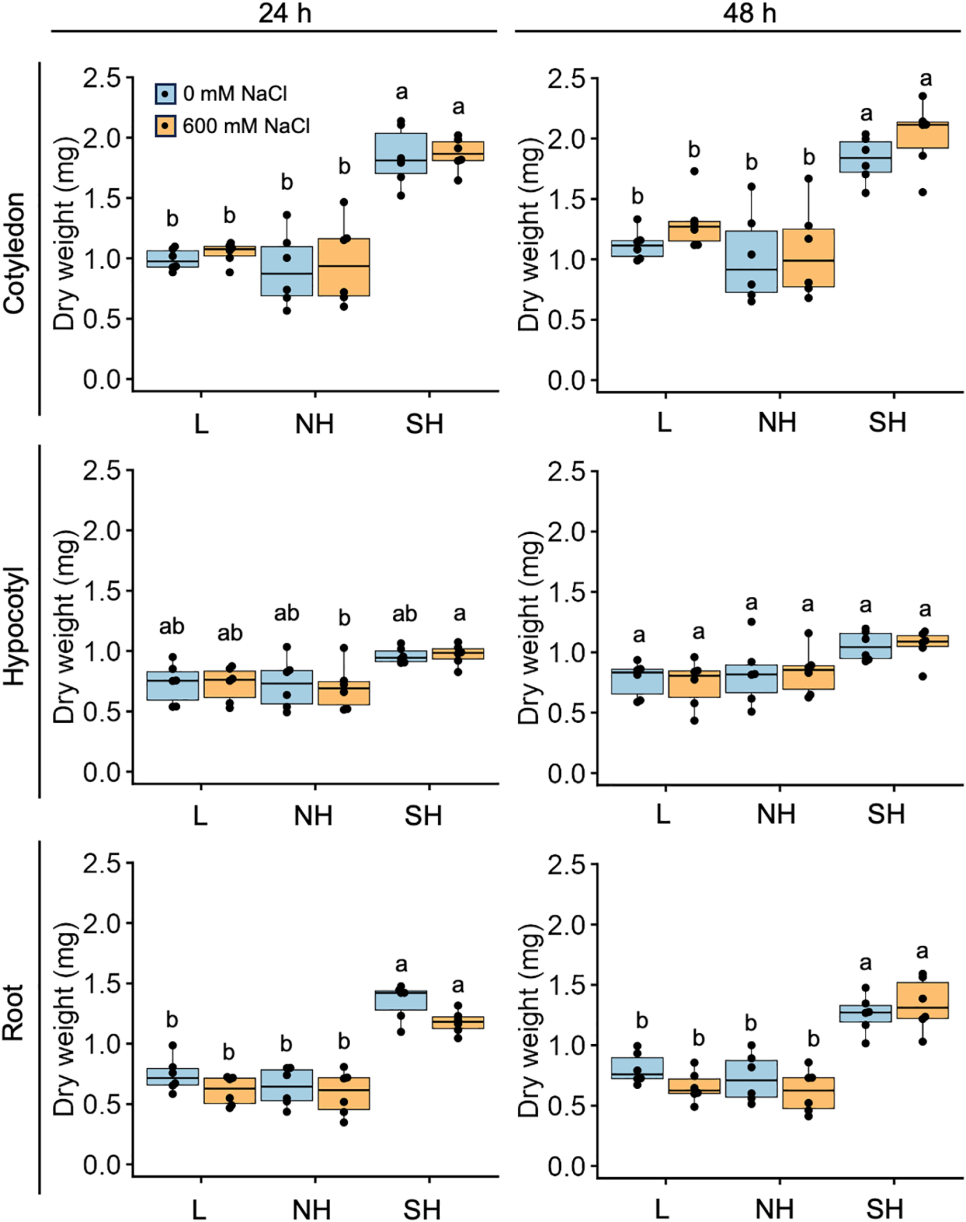
Genotype-specific growth in response to high salinity over time. Ten-day-old seedlings of quinoa inbred lines were treated with 0 or 600 mM NaCl for 24 h (left) and 48 hours (right). Data for lowland (L) lines include Kd, J028, J045, J079, J082, and J122; data for northern highland (NH) lines include J064, J071, J072, J073, J074, and J075; data for southern highland (SH) lines include J054, J094, J096, J099, J100, and J128 (**Supplementary Table 1**). Average dry weight of cotyledons, hypocotyls, and roots of each line is indicated by dots in the box plot. Different letters indicate significant differences among subpopulations by Tukey’s HSD test (*P* < 0.05). Dry weight data for individual lines are shown in **Supplementary Figure 1**.

Next, we investigated the physiological responses of all three subpopulations, as well as the quinoa model line Kd, which belongs to the lowland subpopulation, to high seawater salinity. Based on the results of our previous genetic structure analysis of quinoa inbred lines (Mizuno et al., 2020), we selected six inbred lines containing 100% of the genetic background of each subpopulation from each subpopulation for this experiment (**Supplementary Table 1**). For all three genotypes examined, there was no significant difference in the dry weights of cotyledons, hypocotyls, and roots between seedlings treated or not with 600 mM NaCl at either 24 or 48 h after the salt treatment (**Figures 3**, **4**; **Supplementary Figure 1**), indicating that this treatment did not affect the initial growth of quinoa seedlings of any of the three genotypes. Interestingly, although the 600 mM NaCl treatment did not affect the growth of young Kd seedlings, it caused a rapid upward (hyponastic) leaf movement (**Figure 3**), suggesting that these plants have a physiological response to salt stress.

### 3.3 Quinoa genotype determines aboveground uptake of Na^+^

**Figure 5:**
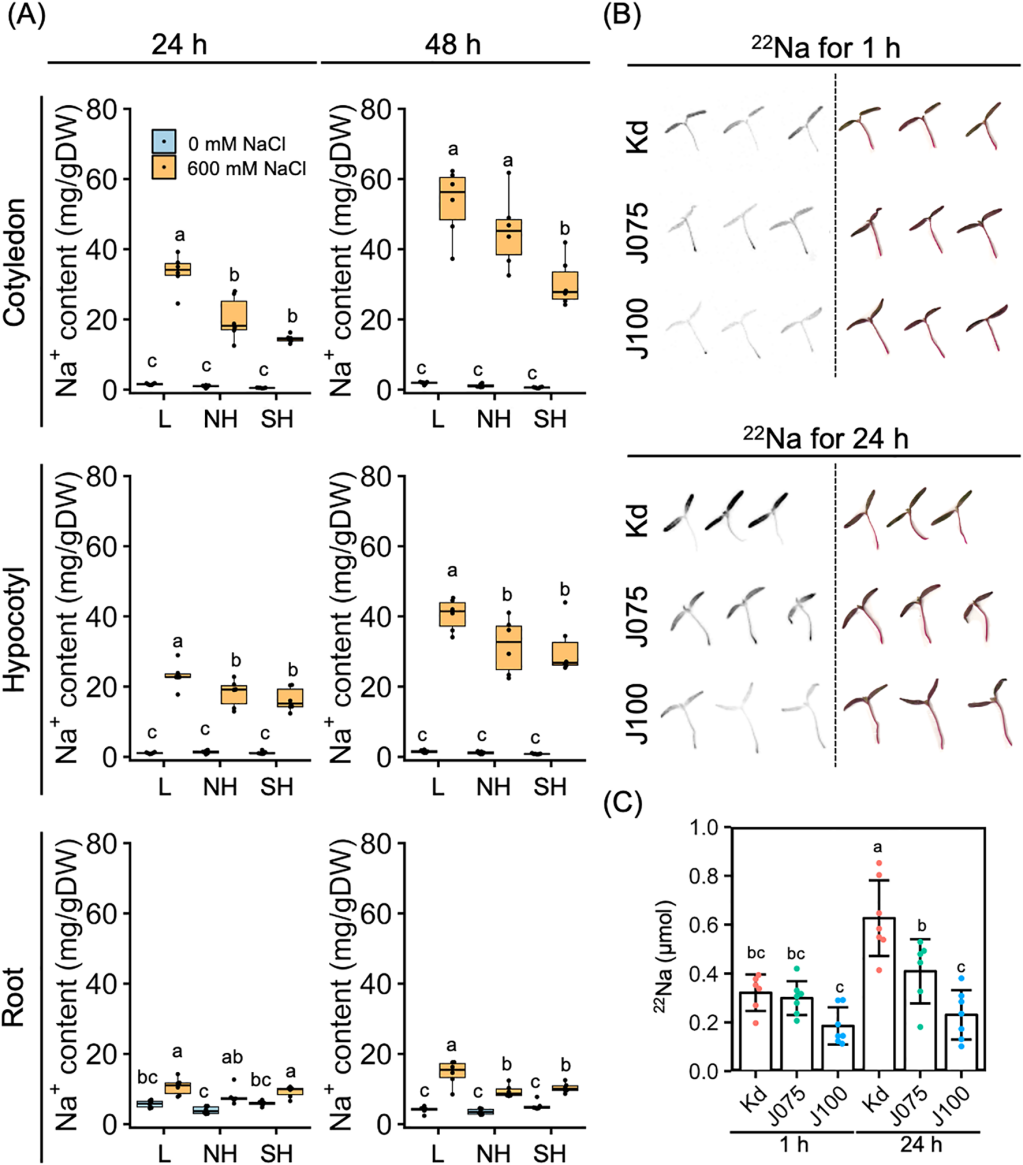
Genotype-specific Na^+^ accumulation and uptake in response to high salinity over time. **(A)** Ten-day-old seedlings of quinoa inbred lines were treated with 0 or 600 mM NaCl for 24 h (left) and 48 h (right). Data for lowland (L) lines include Kd, J028, J045, J079, J082, and J122; data for northern highland (NH) lines include J064, J071, J072, J073, J074, and J075; data for southern highland (SH) lines include J054, J094, J096, J099, J100, and J128 (**Supplementary Table 1**). Average Na^+^ content in cotyledons, hypocotyls, and roots of each line is indicated by dots in the box plot. Different letters indicate significant differences among subpopulations by Tukey’s HSD test (*P* < 0.05). Na^+^ accumulation data for individual lines are shown in **Supplementary Figure 2**. **(B)** Autoradiography (left) and actual photographs (right) of aerial parts of quinoa Kd, J075, and J100 seedlings radiolabeled with ^22^Na. The seedlings were soaked in water containing ^22^Na for 1 and 24 hours. **(C)** ^22^Na content of the aerial part of each quinoa line, calculated from a photostimulated luminescence value and a calibration curve based on the spots (*n* = 6–7 seedlings). Different letters indicate significant differences among inbred lines by Tukey’s HSD test (*P* < 0.05).

We then examined tissue-specific Na^+^ accumulation in young quinoa seedlings treated or not with 600 mM NaCl for 24 and 48 h using six representative inbred lines of each of the three genotypes (**Figure 5A**; **Supplementary Figure 2**; **Supplementary Table 1**). In all salt-treated lines, Na^+^ accumulation tended to be highest in the cotyledons, at intermediate levels in the hypocotyls, and lowest in the roots (**Figure 5A**), indicating that young quinoa seedlings grown in a high-salinity environment accumulate more Na^+^ in the aerial parts than in the roots (**Figure 5A**; **Supplementary Figure 2**). Na^+^ accumulation in aerial parts, especially in the cotyledons, tended to be higher in the lowland lines and lower in the southern highland lines (**Figure 5A**).

To determine whether this difference in Na^+^ accumulation in aerial parts by genotype was due to differences in growth or Na^+^ uptake by genotype, we performed uptake experiments with radiolabeled ^22^Na using Kd, J075, and J100 as three lines with each genotype background (**Figure 5B**). At 24 h after transferring the young seedlings to the ^22^Na-containing medium, we observed significantly higher ^22^Na signals in the aerial parts in the order of Kd, J075, and J100 (**Figures 5B, C**). Thus, Na^+^ uptake in the aerial parts of quinoa was higher in the lowland line (Kd), followed by the northern highland line (J075) and the southern highland line (J100), demonstrating that Na^+^ uptake in the aerial parts of quinoa is determined by the genotype. In addition, it did not induce rapid hyponastic leaf movement at low Na concentrations such as those used in this experiment (**Figure 5B**), supporting the hypothesis that hyponastic leaf movement may play a physiological role in response to high salinity.

### 3.4 High salinity reduces root K^+^ accumulation in all three genotypes

**Figure 6:**
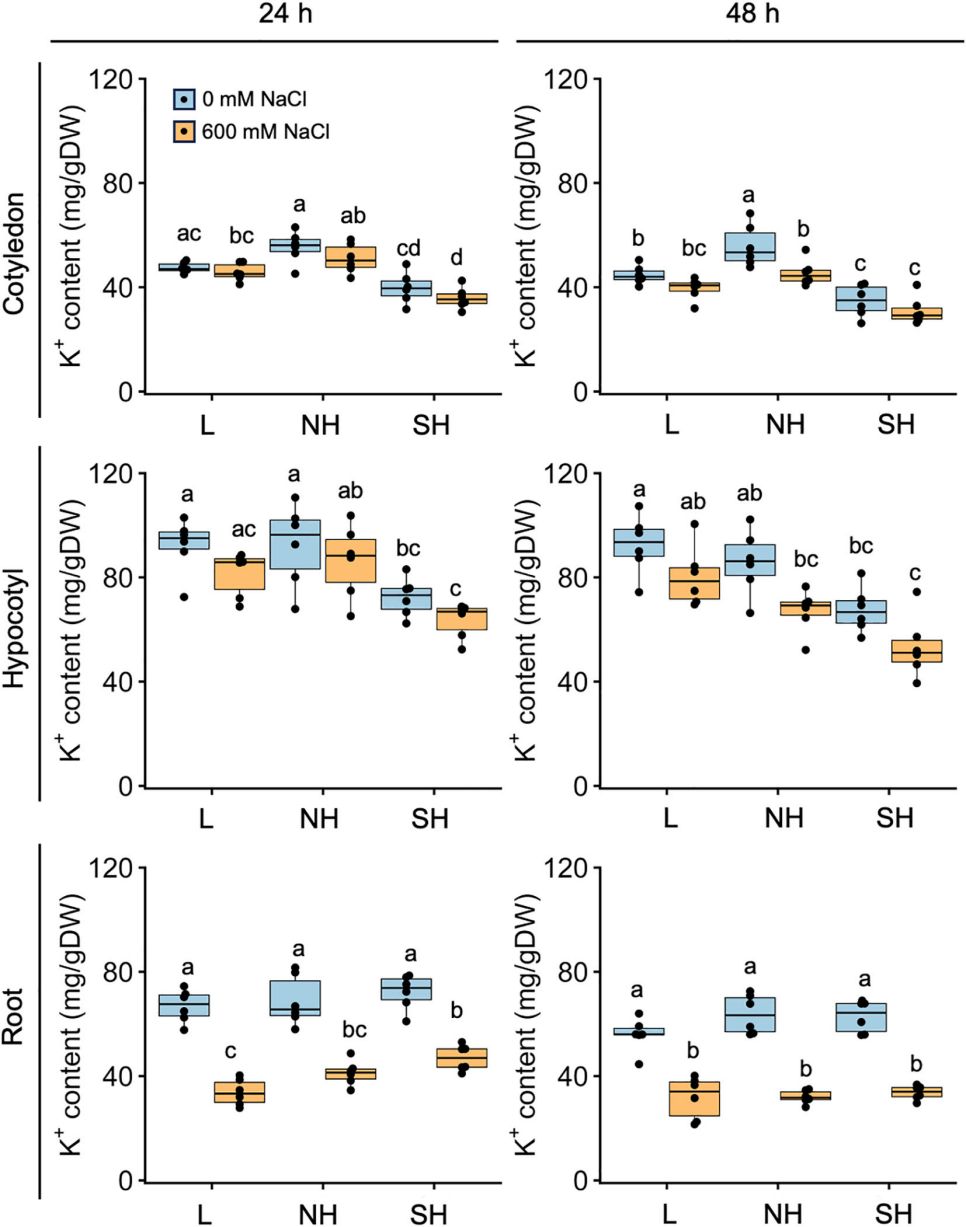
Genotype-specific K^+^ accumulation in response to high salinity over time. Ten-day-old seedlings of quinoa inbred lines were treated with 0 or 600 mM NaCl for 24 h (left) and 48 h (right). Data for lowland (L) lines include Kd, J028, J045, J079, J082, and J122; data for northern highland (NH) lines include J064, J071, J072, J073, J074, and J075; data for southern highland (SH) lines include J054, J094, J096, J099, J100, and J128 (**Supplementary Table 1**). Average K^+^ content in cotyledons, hypocotyls, and roots of each line is indicated by dots in the box plot. Different letters indicate significant differences among subpopulations by Tukey’s HSD test (*P* < 0.05). K^+^ accumulation data for individual lines are shown in **Supplementary Figure 3**.

We further assessed tissue-specific K^+^ accumulation in young quinoa seedlings treated or not with 600 mM NaCl for 24 and 48 h using six representative inbred lines of each of the three genotypes (**Figure 6**; **Supplementary Figure 3**; **Supplementary Table 1**). In cotyledons and hypocotyls, salt treatment tended to slightly reduce K^+^ accumulation in many of the lines (**Figure 6**). Regardless of the salinity conditions, K^+^ accumulation tended to be slightly lower in the aerial parts of southern highland lines than in those of the lowland and northern highland lines (**Figure 6**). By contrast, in roots, salt treatment significantly reduced K^+^ accumulation in all lines examined, and K^+^ accumulation differed little among genotypes (**Figure 6**). The results obtained so far showed that Na^+^ accumulation in the cotyledons of the salt-treated young seedlings is different among the genotypes but that there is no clear difference in Na^+^ and K^+^ accumulation in the roots among the genotypes (**Figures 5**, **6**). We therefore concluded that salt treatment generally increases Na^+^ accumulation and decreases K^+^ accumulation in whole seedlings across all genotypes (**Figures 5**, **6**).

### 3.5 Gene expression profile in response to high salinity varies widely among genotypes

**Figure 7:**
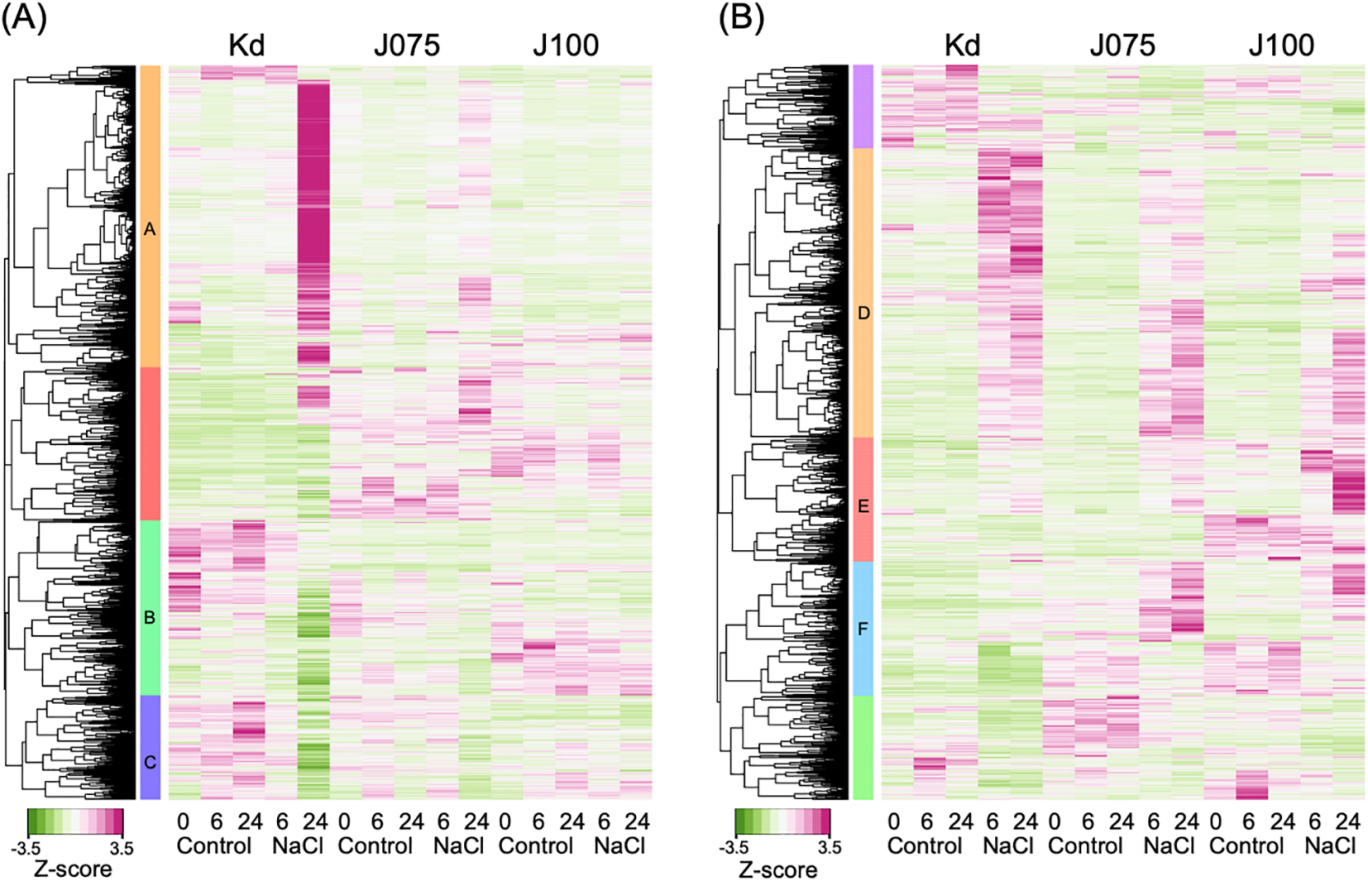
Tissue-specific salt stress–responsive gene expression in genotype representative lines. Hierarchical clustering of 8,303 and 4,121 DEGs in the RNA-seq experiments (|log_2_(FC)| ≥ 1, TPM value > 0, FDR < 0.01) that were identified in cotyledons **(A)** and roots **(B)** of each genotype representative line subjected to either the 6 or 24 h of NaCl treatment compared to the control (0 mM NaCl treatment) (**Supplementary Table 3**). Ten young quinoa seedlings were sampled at each time point of each treatment and replicated in triplicate. Six gene sets, designated clusters A through F, of the nine clusters identified in total were enriched in genes involved in biological processes related to characteristic responses (see **Supplementary Figures 4**–**9** for details). Heat map showing z-scaled TPM of DEGs.

To investigate the effect of high salinity on gene expression in young quinoa seedlings, we analyzed the gene expression profiles of cotyledons and roots after 0, 6, and 24 h of treatment with or without 600 mM NaCl (**Figure 7**). We used Kd (Yasui et al., 2016) as a genome-sequenced representative lowland inbred line, J075 (Kobayashi et al., 2024) as a genome-sequenced representative northern highland inbred line, and J100 (Kobayashi et al., 2024) as a genome-sequenced representative southern highland inbred line (**Figure 7**). In the RNA-seq experiment, we identified 8,303 and 4,121 DEGs (|log_2_(FC)| ≥ 1, TPM value > 0, FDR < 0.01) in response to high salinity in at least one sampling in cotyledons and roots of each line, respectively (**Figure 7**; **Supplementary Table 3**). We then performed hierarchical clustering and GO enrichment analysis of these genes. In cotyledons, one gene set, designated cluster A, was enriched in genes involved in biological processes related to cell wall organization (**Figure 7A**; **Supplementary Figure 4**; **Supplementary Table 4**). Two other gene sets, designated clusters B and C, were enriched in genes involved in biological processes related to photosynthesis and cellular response to hypoxia (**Figure 7A**; **Supplementary Figures 5**, **6**; **Supplementary Table 4**). These results indicate that, in cotyledons of the salt-stressed lowland Kd line, the expression of genes related to cell wall composition is up-regulated, while the expression of genes related to photosynthesis and oxygen deprivation response is down-regulated. In roots, one gene set, designated cluster D, was enriched in genes involved in biological processes related to responses to water deprivation and ABA (**Figure 7B**; **Supplementary Figure 7**; **Supplementary Table 5**). Two additional gene sets, designated clusters E and F, were enriched in genes involved in biological processes related to cellular response to hypoxia, defense response to bacteria, and hydrogen peroxide catabolic process (**Figure 7B**; **Supplementary Figures 8**, **9**; **Supplementary Table 5**). To validate the RNA-seq expression data, we performed RT-qPCR analyses on 18 inbred lines−six from each genotype−under the same conditions. We analyzed a putative *quinoa xyloglucan endotransglucosylase/hydrolase 23* (*CqXTH23*) and a putative *quinoa cellulose synthase 1* (*CqCESA1*) genes included in cluster A and a putative *quinoa chaperon protein ClpB1* (*CqCLPB1*), a putative *quinoa zinc finger protein ZAT12* (*CqZAT12*), and a putative *quinoa peroxidase PER4* (*CqPER4*) genes included in clusters E and F, and verified the results of these RNA-seq analyses (**Supplementary Figures 10**, **11**). This consistency supports the reliability of the expression patterns identified across these genotypes and conditions. Taken together, these results indicate that all lines tested, regardless of the genotype, showed increased expression of genes involved in responses to water deprivation and ABA in their roots under high salinity conditions. Interestingly, in the roots of the highland lines J075 and J100, salt application specifically resulted in a significant increase in the expression of genes related to the hypoxia response, defense response against bacteria, and reactive oxygen species (ROS) pathways.

### 3.6 *CqHKT1* and *CqSOS1* gene expression levels are genotype dependent

So far, we have analyzed gene expression responses to high salinity, but salt stress–tolerant plants often do not show significant changes in the expression of key genes involved in the salt stress response due to constitutive expression of key genes such as *HKT1* and *SOS1* (Taji et al., 2004; Katschnig et al., 2015). The quinoa sodium transporter *CqHKT1* gene has been reported to have a root-expressed *CqHKT1;1* and a shoot-expressed *CqHKT1;2* (Böhm et al., 2018). *CqSOS1* is also conserved in the quinoa genome and is mainly expressed in roots (Maughan et al., 2009). The expression of these genes remained unchanged in quinoa subjected to salt stress treatment (Maughan et al., 2009; Böhm et al., 2018). The results of these reports are largely consistent with those of our RNA-seq analysis (**Supplementary Figure 12**). To date, *in vitro* functional analysis has been performed for CqHKT1;1 and CqHKT1;2 (Böhm et al., 2018), but no functional analysis results have been reported for CqSOS1. However, a comparison of the predicted three-dimensional structures shows that CqHKT1 and CqSOS1, which are homologs of quinoa HKT1 and SOS1, both have high homology with the Arabidopsis homologous proteins, suggesting functional similarity (**Supplementary Figure 13**).

To explore the genetic variation in the quinoa inbred lines examined in this study, we employed resequencing to analyze 18 of the quinoa inbred lines (**Supplementary Table 1**). Genomic analysis showed that the *CqHKT1;1* gene in lines J072, J073, and J075 had a single nucleotide insertion in the first exon, which shifted the reading frame (**Supplementary Figure 14**). Expression of this disrupted gene was completely undetectable (**Supplementary Figure 15**). In lines J064 and J071, a 6.9-kb deletion was found in the 5′ flanking region of the *CqSOS1* gene (**Supplementary Figure 16**), and almost no gene expression was observed (**Supplementary Figure 15**). By contrast, the other lines examined showed full-length gene expression with no mutations in or near the *CqHKT1;1* or *CqSOS1* genes (**Supplementary Figures 17**–**19**).

**Figure 8:**
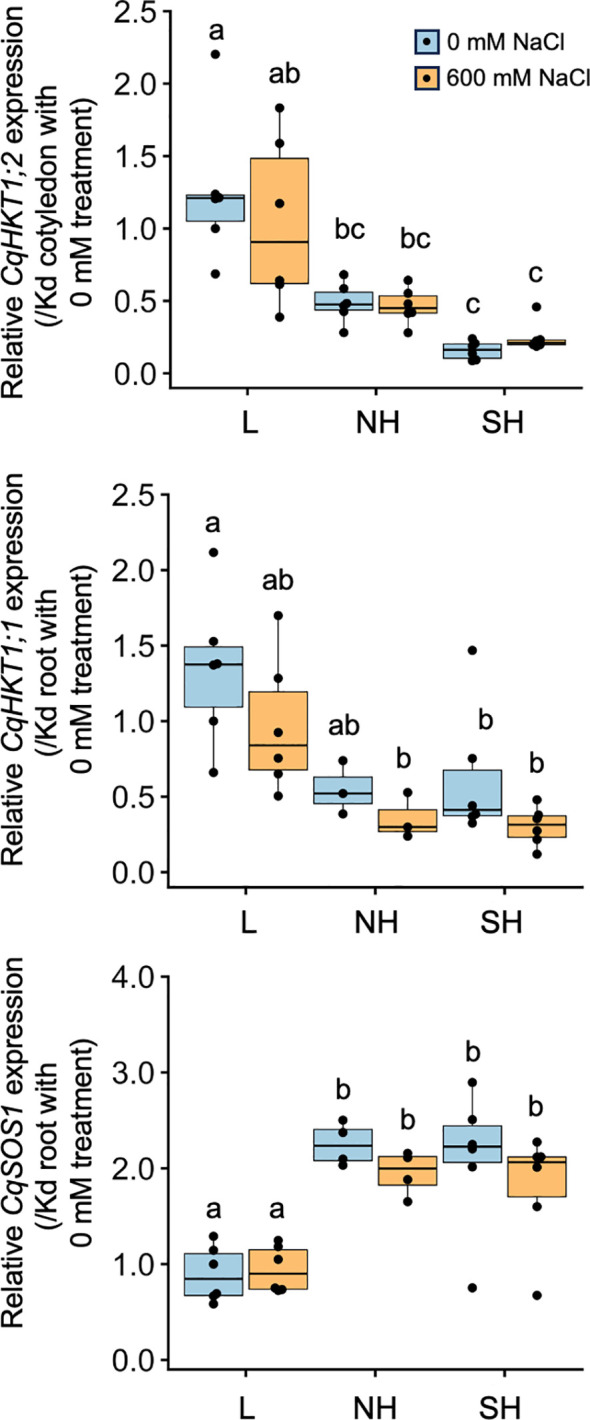
Genotype-specific expression of transporter genes in response to high salinity. Ten-day-old seedlings of quinoa inbred lines were treated with 0 or 600 mM NaCl for 24 h. Gene expression in cotyledons was examined for *CqHKT1;2*, which is mainly expressed in cotyledons, and gene expression in roots was examined for *CqHKT1;1* and *CqSOS1*, which are mainly expressed in roots. The transcript levels of *CqHKT1;2*, *CqHKT1;1*, and *CqSOS1* genes were normalized to those of *CqUBQ10* as an internal control gene. Average expression levels of *CqHKT1;1, CqHKT1;2*, and *CqSOS1* are shown as dots in the box plots relative to the gene expression levels in each corresponding tissue in the non-salt-treated young Kd seedlings. Data for lowland (L) lines include Kd, J028, J045, J079, J082, and J122; data for northern highland (NH) lines include J064, J071, J072, J073, J074, and J075; and data for southern highland (SH) lines include J054, J094, J096, J099, J100, and J128 (**Supplementary Table 1**). The *CqHKT1;1* genes in J072, J073, and J075 and the *CqSOS1* genes in J064 and J071 had an adenine insertion in the first exon and a 6.9-kb deletion in the 5′ region of the gene’s coding region and the corresponding transcripts were not full-length or functional; gene expression data for these genes are not included. Different letters indicate significant differences among subpopulations based on a Tukey’s HSD test (*P* < 0.05). Relative expression levels for individual lines are shown in **Supplementary Figure 15**.

RT-qPCR analysis revealed that *CqHKT1;2* in cotyledons and *CqHKT1;1* and *CqSOS1* in roots did not show significant changes in gene expression in response to high salinity in many of the lines examined, but there were significant differences in expression levels among genotypes with and without salt treatment (**Figure 8**; **Supplementary Figure 15**). However, the expression levels of these genes tended to be more genotype-dependent in *CqHKT1;2* and *CqSOS1*, and less so in *CqHKT1;1* (**Figure 8**; **Supplementary Figure 15**). In most cases, *CqHKT1;1* and *CqHKT1;2* were expressed higher in lowland lines than in highland lines, whereas *CqSOS1* was expressed higher in southern and northern highland lines than in lowland lines (**Figure 8**; **Supplementary Figure 15**). By contrast, it appears that genotype-dependent gene expression pattern was not clearly observed for the expression of K^+^ transporter genes (**Supplementary Figures 20**, **21**), consistent with the property of maintaining high K^+^ concentrations in the aerial part even under high salinity conditions, regardless of genotype (**Figure 6**). In addition, many sequence polymorphisms were observed around the promoter regions of the *CqHKT1;1*, *CqHKT1;2*, and *CqSOS1* genes between lowland and highland quinoa lines (**Supplementary Figures 17**–**19**), strengthening the hypothesis that the expression levels of these transporter genes are genotype dependent. Given that constitutive gene expression levels of transporters are associated with Na^+^ accumulation and salt tolerance (Oh et al., 2009; Jha et al., 2010), these results support the notion that the function and activity of the transporters are defined by genotype in quinoa.

### 3.7 CqHKT1 and CqSOS1 mediate Na^+^ exclusion in quinoa

**Figure 9:**
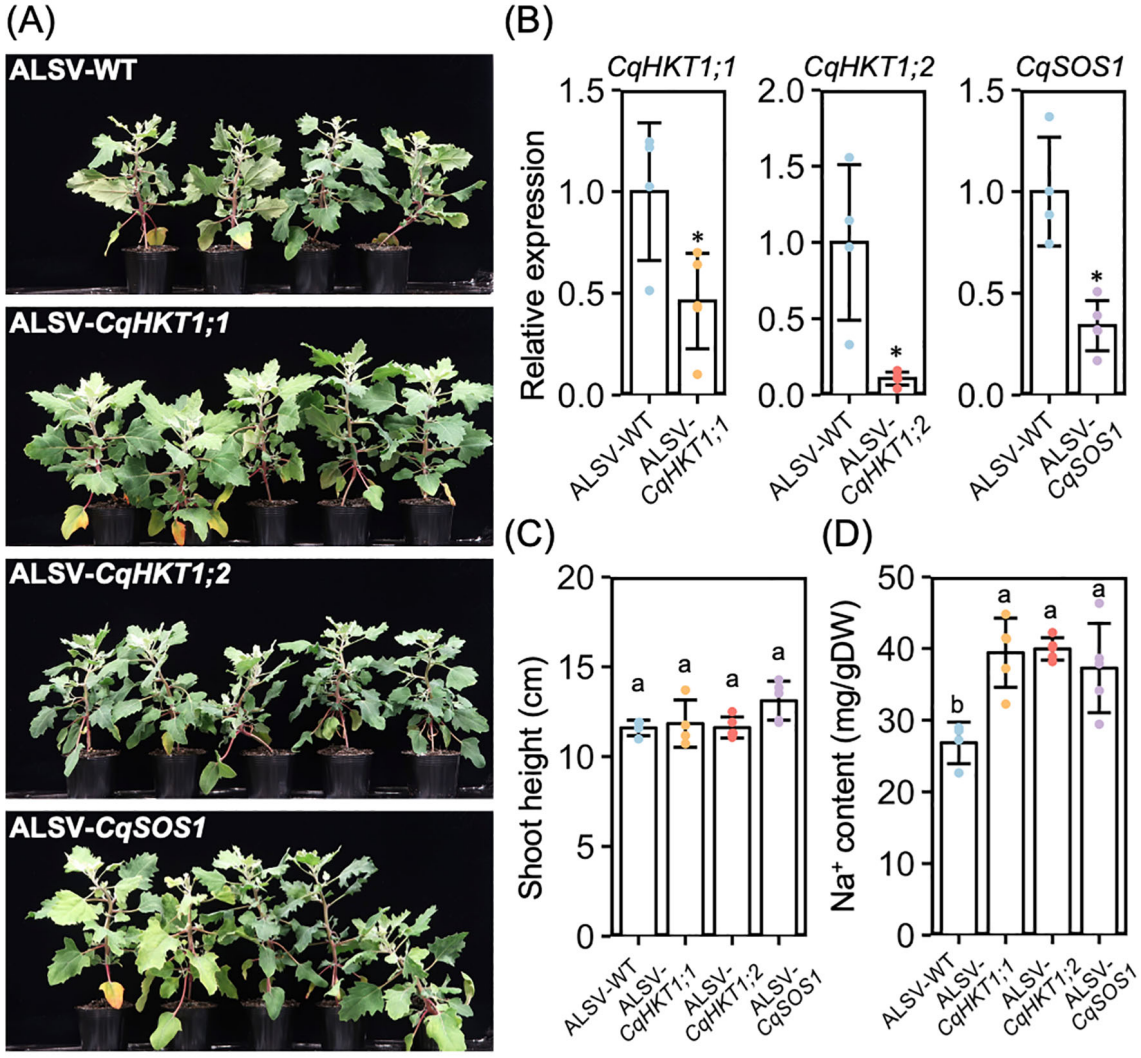
Knockdown of *CqHKT1;1*, *CqHKT1;2* or *CqSOS1* increased Na^+^ content in the uninoculated upper leaves of salt-treated plants. **(A)** Representative images of plants inoculated with ALSV-WT, ALSV-CqHKT1;1, ALSV-CqHKT1;2, or ALSV-CqSOS1 for 10 days and then treated with 300 mM NaCl for 21 days. **(B)** RT-qPCR quantification of *CqHKT1;1, CqHKT1;2* and *CqSOS1* transcripts in the uninoculated upper leaves of plants inoculated with the indicated inocula. Data were normalized to the *CqUBQ10* levels and are presented as means ± SD (*n* = 4 for ALSV-WT; *n* = 5 for ALSV-each target gene). **(C)** Plants were treated with 300 mM NaCl 10 days after inoculation, and shoot height was measured 21 days later. **(D)** Na^+^ content of the lowest uninoculated upper leaves of virus vector inoculated plants on day 21 of NaCl treatment. Data show mean ± SD (*n* = 4 in ALSV-WT and *n* = 5 in ALSV-each target gene). Asterisks and different lowercase letters indicate significant difference at *P* < 0.05, as determined using Student’s *t*-test and Tukey’s HSD test.

To determine whether the CqHKT1 and CqSOS1 transporters are involved in Na^+^ transport in quinoa, we suppressed the gene expression of these transporters using the VIGS method with an ALSV vector, the only method currently available to analyze endogenous gene function in quinoa (Ogata et al., 2021) (**Figure 9**). Because this ALSV-VIGS experimental system requires virus inoculation of leaves with epidermal bladder cells and sampling of uninoculated upper leaves with epidermal bladder cells, the transport ability of the transporters was analyzed in older quinoa plants, which have true leaves with epidermal bladder cells, rather than in seedlings, which lack epidermal bladder cells. In addition, a model quinoa line, the lowland line Kd, was used in this experiment. This is because lowland lines tend to accumulate more Na^+^ in the aerial parts under high salinity conditions, unlike southern highland lines, and are therefore more suitable for analyzing the function of these transporters. RT-qPCR analysis revealed that *CqHKT1;1*, *CqHKT1;2*, and *CqSOS1* expression was significantly down-regulated in the uninoculated upper leaves of the plants inoculated with ALSV-CqHKT1;1, ALSV-CqHKT1;2, and ALSV-CqSOS1, respectively, compared with those inoculated with ALSV-WT in each experiment (**Figure 9B**). Knockdown of *CqHKT1;1*, *CqHKT1;2*, and *CqSOS1* had no significant effect on plant height (**Figures 9B, C**) but increased the Na^+^ content in the uninoculated upper leaves of salt-treated plants by 56%, 47%, and 55%, respectively, indicating that reduced expression of these transporter genes results in increased salt accumulation in plants (**Figure 9D**). Considering that these transporters generally function to control Na^+^ homeostasis through long-distance transport of Na^+^ in a variety of plants (Ali et al., 2021; Gámez-Arjona et al., 2024), these results demonstrate that *CqHKT1;1*, *CqHKT1;2*, and *CqSOS1* function in Na^+^ exclusion in the long-distance transport system under high salinity conditions in quinoa.

## 4 Discussion

Using our previously reported VIGS application with the ALSV vector (Ogata et al., 2021), we show for the first time that the quinoa transporters CqHKT1;1, CqHKT1;2, and CqSOS1 contribute to Na^+^ exclusion in quinoa plants (**Figure 9**). Considering that most of the previous findings of transporters involved in salt transport in quinoa were based on genomic and gene expression analysis (Zou et al., 2017; Shi and Gu, 2020), the demonstration of quinoa transporter function using VIGS in this study is an important step towards understanding the salt tolerance mechanism in quinoa. However, much remains to be resolved. There are no significant differences in Na^+^ or K^+^ accumulation or growth between the roots of northern highland lines that harbor or lack functional *CqHKT1;1* or *CqSOS1* genes (**Figures 3**–**6**; **Supplementary Figures 1**–**3**). By contrast, no northern highland lines lack functional versions of both *CqHKT1;1* and *CqSOS1* functioning in the roots (**Supplementary Figures 14**–**16**). These observations suggest that, in northern highland quinoa lines, it may be sufficient to have either *CqHKT1;1* or *CqSOS1*, or that other genes may be involved in Na^+^ exclusion in leaves. In leaves, the inward rectifier CqHKT1;2 has been reported to play a key role in Na^+^ loading of bladder cells under salt stress (Böhm et al., 2018), but its primary role in quinoa needs to be clarified in the context of findings from homologous genes in other plants. In this study, we show that both CqHKT1;2, which functions mainly in leaves, and CqHKT1;1 and CqSOS1, which function mainly in roots, are involved in Na^+^ exclusion through gene repression (**Supplementary Figure 12**). The roles of the transporters involved in salt tolerance in quinoa and the properties that make these transporters superior to those of other plants that are less salt tolerant than quinoa remain to be elucidated. Thus, the present study provides a basis for solving these important mysteries of the salt tolerance mechanism.

We found that young quinoa seedlings at a growth stage lacking epidermal bladder cells grow as well as or better than non-salt-treated control plants under 600 mM NaCl (**Figures 2**–**4**; **Supplementary Figure 1**), indicating that the salt tolerance of young quinoa seedlings is far greater than that of model and crop plants (Nanjo et al., 1999; Zapata et al., 2017). For example, Arabidopsis plants fall over within 30 min of exposure to 600 mM NaCl (Nanjo et al., 1999), and lettuce and pepper seedlings show suppressed growth after 24 h of exposure to 100 mM NaCl (Zapata et al., 2017). Given that the Na^+^ influx into plant cells causes K^+^ efflux, a phenomenon involved in many physiological processes, disrupting K^+^ homeostasis (Wang et al., 2009; Demidchik, 2014; Pottosin and Shabala, 2014; Assaha et al., 2017), the ability of quinoa to maintain high K^+^ levels even under high-salinity conditions may contribute to its high salt tolerance (**Figure 6**). In fact, aboveground K^+^ contents in quinoa seedlings do not change significantly under high-salt conditions (**Figure 6**). More interestingly, the differences among quinoa genotypes in K^+^ accumulation and K^+^ transporter gene expression under high-salt conditions are smaller than those in Na^+^ accumulation and Na^+^ transporter gene expression (**Figures 5**, **6**, **8**; **Supplementary Figures 15, 20, 21**). These observations support the idea that quinoa may have a sophisticated control system that prioritizes K^+^ retention to maintain growth and metabolic activity. Recent physiological and biochemical findings from five highland quinoa lines suggest that leaf osmotic regulation, K^+^ retention, Na^+^ exclusion, and ion homeostasis are the major physiological mechanisms that confer salt stress tolerance to highland quinoa lines (Cai and Gao, 2020). Based on these results, we believe further research focusing on ion transport is necessary to elucidate the salt tolerance mechanism in quinoa.

We performed time-course tissue-specific transcriptome analysis of young seedlings of quinoa inbred lines with three genetic backgrounds (northern highland, southern highland and lowland) under high salt concentrations and identified interesting DEGs in three quinoa lines with different genetic backgrounds and different salt response phenotypes (**Figures 7**; **Supplementary Figures 4**–**12**; **Supplementary Tables 3**–**5**). Our study differs from previous transcriptomic studies of salt stress responses in quinoa (Shi and Gu, 2020; Vita et al., 2021; Luo et al., 2022) in that we used a genome-sequenced inbred line and young seedlings, and we provided a more specific and comprehensive overview of DEGs in salt stress responses in quinoa. Our study clearly showed that cell wall-related genes are highly expressed in leaves with high Na^+^ accumulation (**Figure 7A**; **Supplementary Figures 4**–**6**; **Supplementary Table 4**), consistent with previous observations that cell wall remodeling occurs under salt stress in the other plants (Shen et al., 2014; Wang et al., 2019; Colin et al., 2023). In roots, the expression of genes associated with the ABA pathway and water deprivation response was up-regulated regardless of the genotype, while the expression of genes associated with the hypoxia response, defense response to biotic stress, and the ROS pathway was up-regulated in the highland lines, especially in the southern highland lines (**Figure 7B**; **Supplementary Figures 7**–**9**; **Supplementary Table 5**). The results of these GO analyses suggest that both hypoxia and salt stress induce common defense responses involved in Ca^2+^ signaling, membrane polarization, and ROS metabolic process (Wang et al., 2017; Cackett et al., 2022), and that these responses differ between highland and lowland lines, which respond differently to high salt. Given that transcriptome analysis under salt treatment using salt-tolerant (QQ056) and salt-sensitive (37TES) lowland quinoa lines identified DEGs involved in the defense response to biotic stress (Shi and Gu, 2020), these findings support the notion that important factors determining salt tolerance in quinoa are involved in Ca^2+^ signaling, membrane localization, and ROS pathways common to these defense mechanisms. These observed differences in gene expression between the roots of the lowland quinoa line Kd, which exhibit striking increases in Na^+^ concentration in the aerial parts following high salt treatment, and southern highland quinoa J100, which exhibit lower increases in Na^+^ concentration in the aerial parts following high salt treatment, are expected to provide clues into the mechanisms underlying the high salinity response in quinoa.

We further demonstrated that quinoa genotypes differ significantly in their tendency to accumulate Na^+^ in the aerial parts of the plant under high salinity conditions (**Figures 2**, **5**; **Supplementary Figure 2**). This is the first report on the relationship between genotype and salt accumulation in quinoa using inbred lines based on genomic analysis, and is consistent with a previous observation that, among the lines examined, the genotypes originated from the Bolivian Altiplano had the lowest levels of sodium in leaves under saline conditions (Shabala et al., 2013). Under high-salinity conditions, the lowland quinoa lines accumulated more Na^+^ in the aerial parts, whereas the southern highland lines did not accumulate much Na^+^ in the aerial parts (**Figures 2**, **5**; **Supplementary Figure 2**). We also showed that the difference in Na^+^ accumulation among these genotypes under high-salinity conditions is due to the differences in the aboveground uptake of Na^+^ between highland and lowland lines under high-salinity conditions (**Figure 5B**). In addition, many sequence polymorphisms around the promoter regions of *CqHKT1;1*, *CqHKT1;2*, and *CqSOS1* were observed between lowland and highland quinoa lines (**Supplementary Figures 17**–**19**). The lowland and southern highland lines have both root transporters, CqHKT1;1 and CqSOS1, whereas five of the six northern highland lines have only one of the two root transporters (**Supplementary Figure 15**), supporting the notion that only functional transporters were transmitted from the northern highlands to the southern highlands and lowlands. This may be related to the previous observation that southern highland and lowland quinoa seeds were able to germinate in 600 mM salt water, but northern highland quinoa seeds were unable to germinate (Mizuno et al., 2020). These results therefore suggest that the Na^+^ transporter–mediated phenotype involved in quinoa Na^+^ exclusion is closely related to quinoa genotype, consistent with a previous genome-based hypothesis that domestication first occurred in the northern highlands of the Altiplano (Mizuno et al., 2020).

## Data Availability

The datasets presented in this study can be found in online repositories. The names of the repository/repositories and accession number(s) can be found below: https://www.ddbj.nig.ac.jp/, PRJDB18326 and PRJDB18355.
