# Supplementary material for: CqHKT1 and CqSOS1 mediate genotype-dependent Na+ exclusion under high salinity conditions in quinoa
Source: Front Plant Sci. 2025 Jun 18;16:1597647. doi: 10.3389/fpls.2025.1597647 (PMC12213900; doi:10.3389/fpls.2025.1597647)
Supplement: Supplementary file 1 [file DataSheet1.pdf]

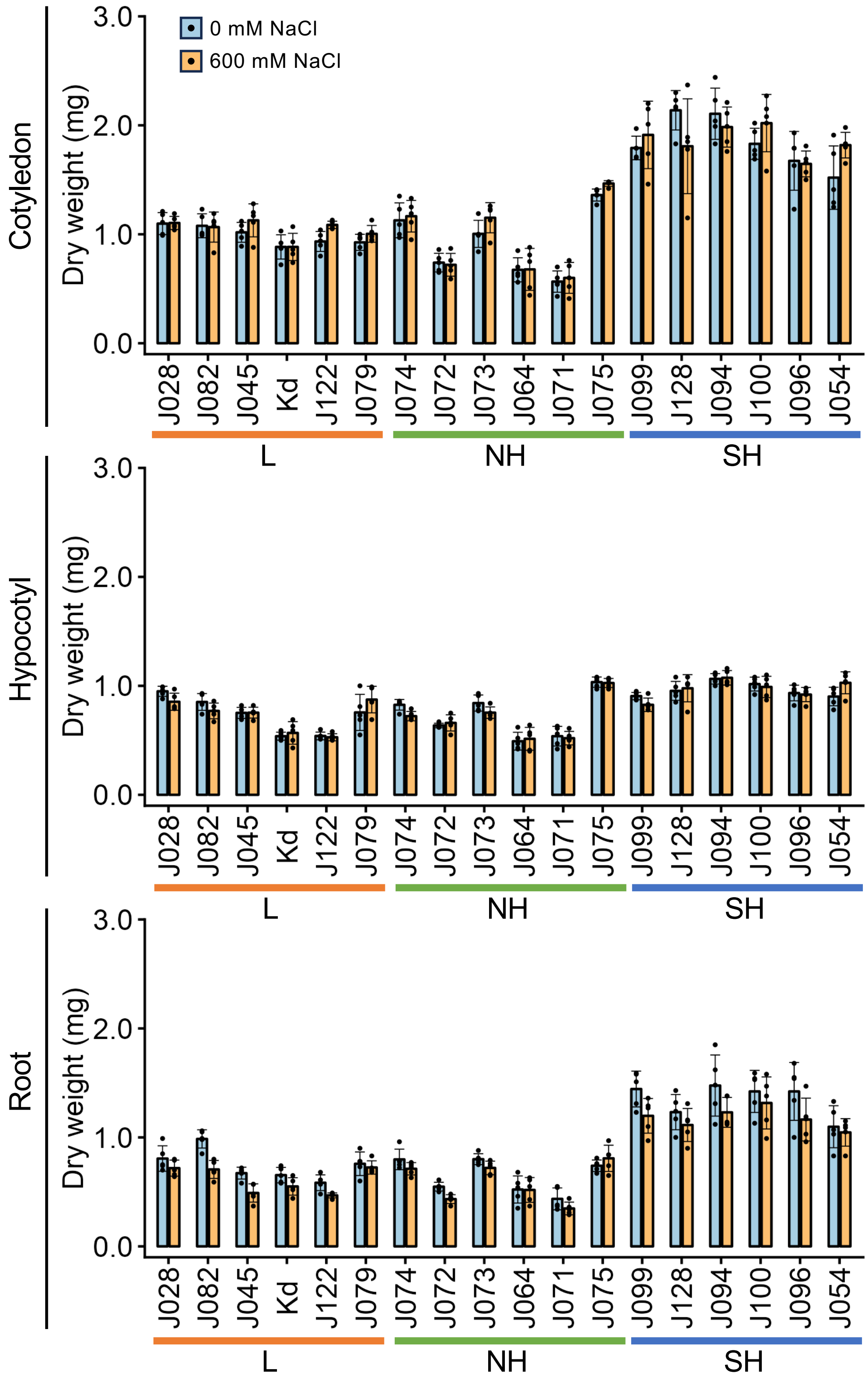

Supplementary Figure 1. Growth of seedlings of representative quinoa inbred lines in response to high salinity over time. Ten-day-old seedlings of quinoa inbred lines were treated with 0 or 600 mM NaCl for 24 h. L, lowland lines; NH, northern highland lines; SH, southern highland lines. To facilitate understanding of the relationship with Na^+ accumulation, the lines within each genotype are arranged from left to right in order of Na^+ content in the cotyledons, as shown in Supplementary Figure 2. Data show mean ± SD of cotyledon, hypocotyl, and root dry weights for each line (n = 5).

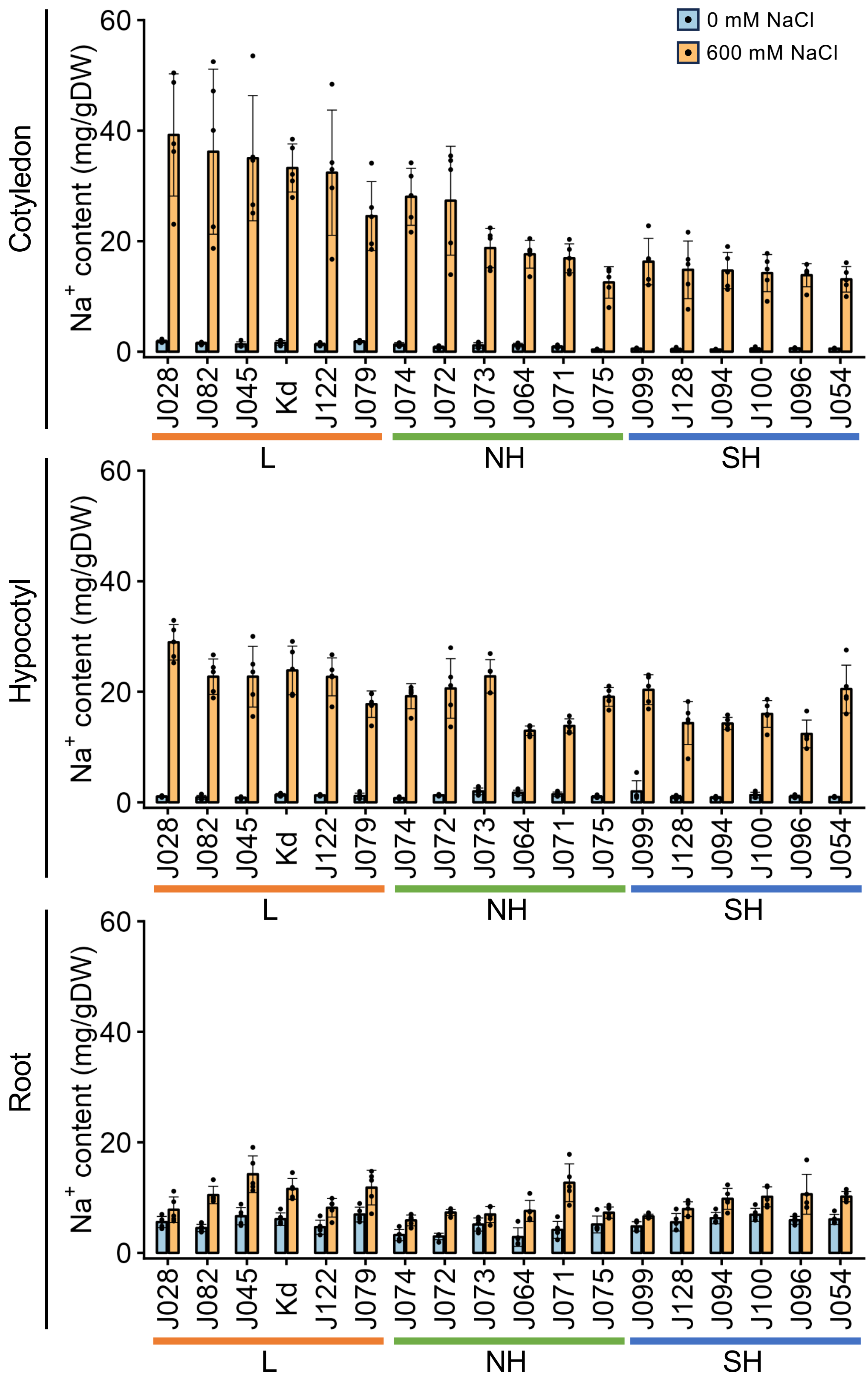

Supplementary Figure 2. Na^+ accumulation in seedlings of representative quinoa inbred lines in response to high salinity over time. Ten-day-old seedlings of quinoa inbred lines were treated with 0 or 600 mM NaCl for 24 h. L, lowland lines; NH, northern highland lines; SH, southern highland lines. To facilitate understanding of the relationship with Na^+ accumulation, the lines within each genotype are arranged from left to right in order of Na^+ content in the cotyledons. Data show mean ± SD of Na^+ content in the cotyledon, hypocotyl, and root of each line ($n = 5$).

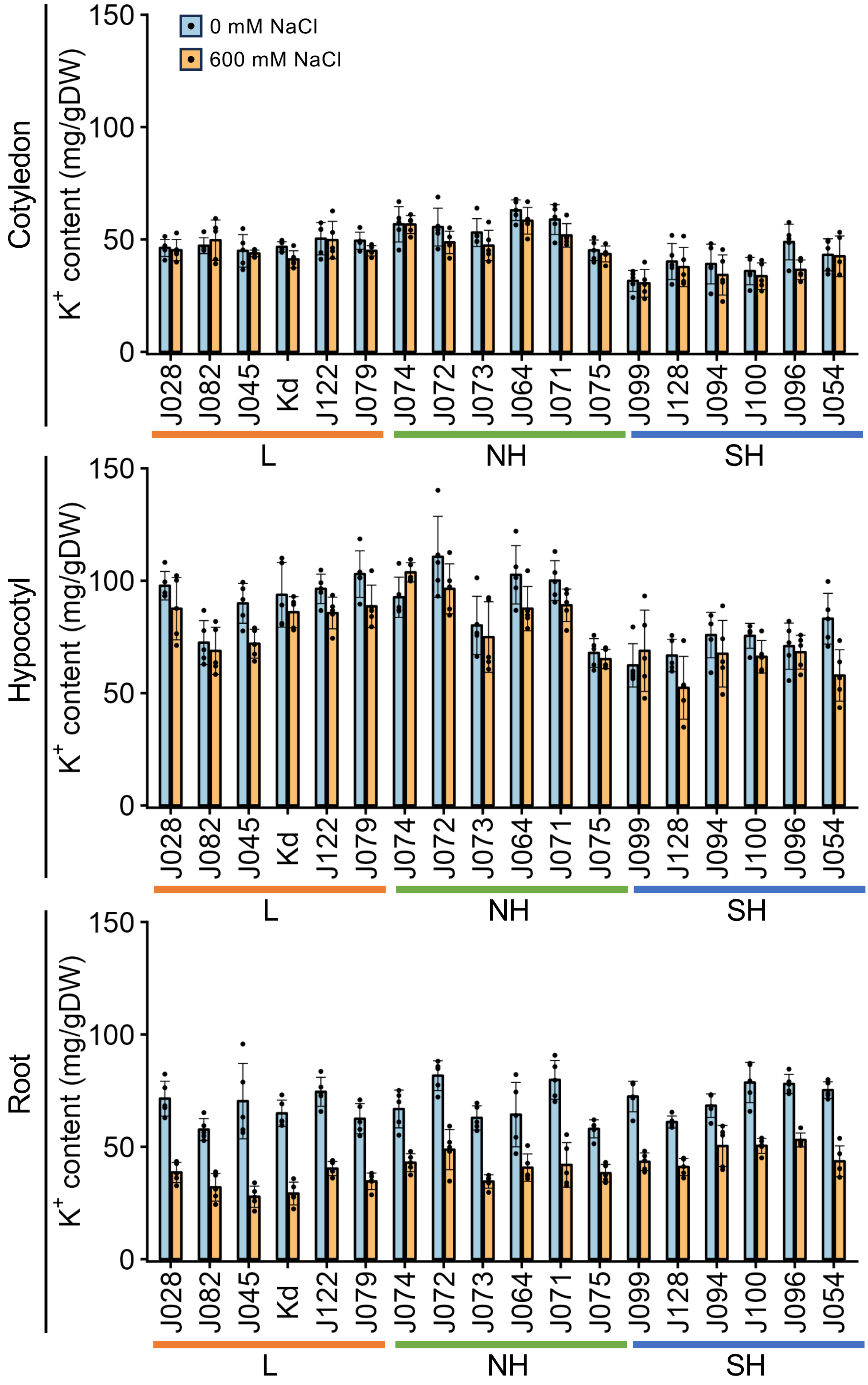

Supplementary Figure 3. K^+ accumulation in seedlings of representative quinoa inbred lines in response to high salinity over time. Ten-day-old seedlings of quinoa inbred lines were treated with 0 or 600 mM NaCl for 24 h. L, lowland lines; NH, northern highland lines; SH, southern highland lines. To facilitate understanding of the relationship with Na^+ accumulation, the lines within each genotype are arranged from left to right in order of Na^+ content in the cotyledons, as shown in Supplementary Figure 2. Data show mean ± SD of K^+ content in the cotyledon, hypocotyl, and root of each line (n = 5).

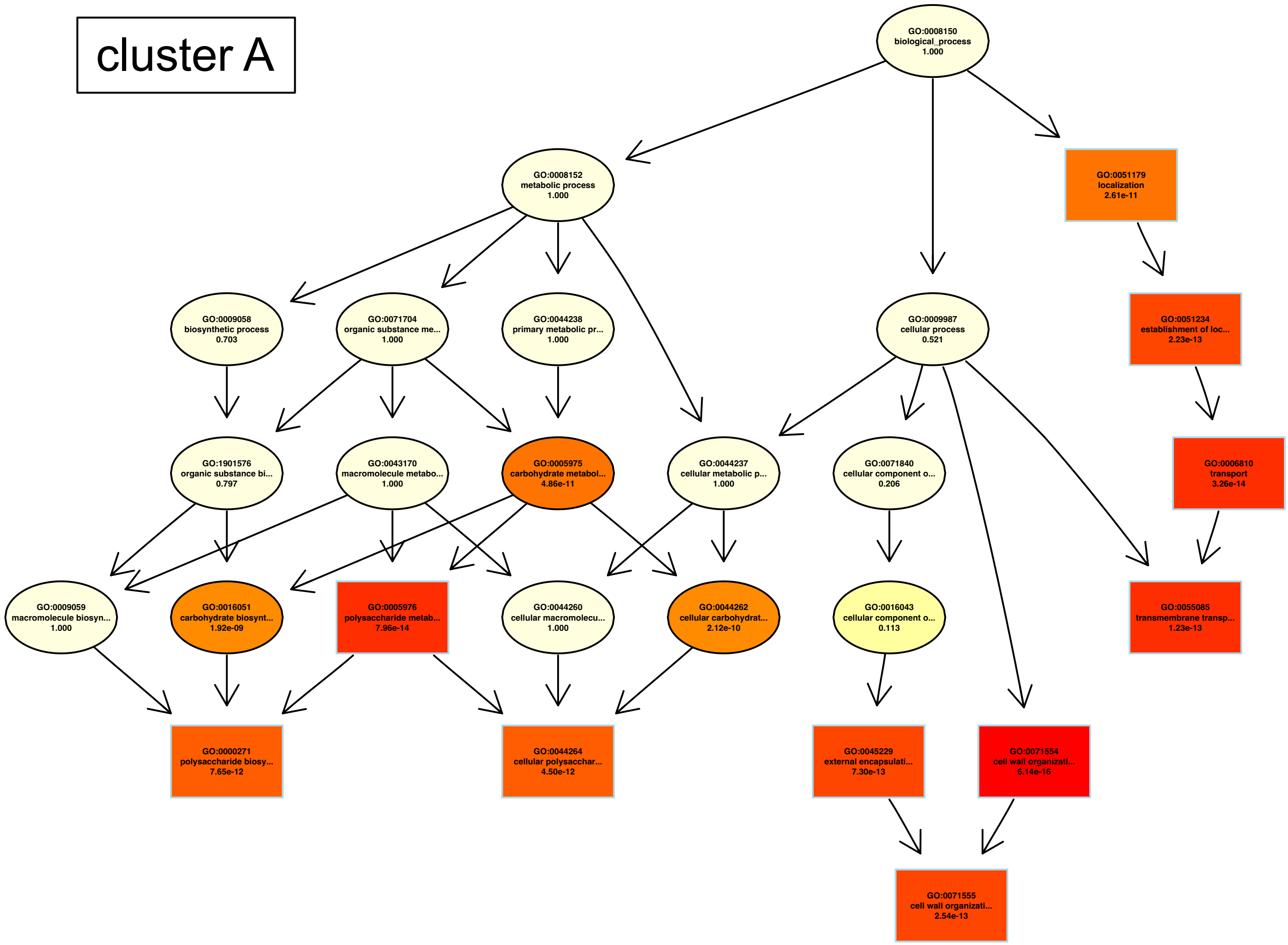

Supplementary Figure 4. High salinity induces the expression of genes related to cell wall organization in the aerial parts of quinoa seedlings of the lowland line, which accumulates Na^+ in the aerial parts. GO term enrichment analysis of cluster A genes (Figure 7A), which are induced to a greater extent under high salt treatment in the cotyledons of young Kd seedlings than in those of J075 and J100. Each enriched biological process GO term is shown in the directed acyclic graphical model. The box indicates the 10 most enriched terms out of the 50 GO terms shown in Supplementary Table 4. Different colors represent different degrees of enrichment significance, with darker colors indicating higher significance.

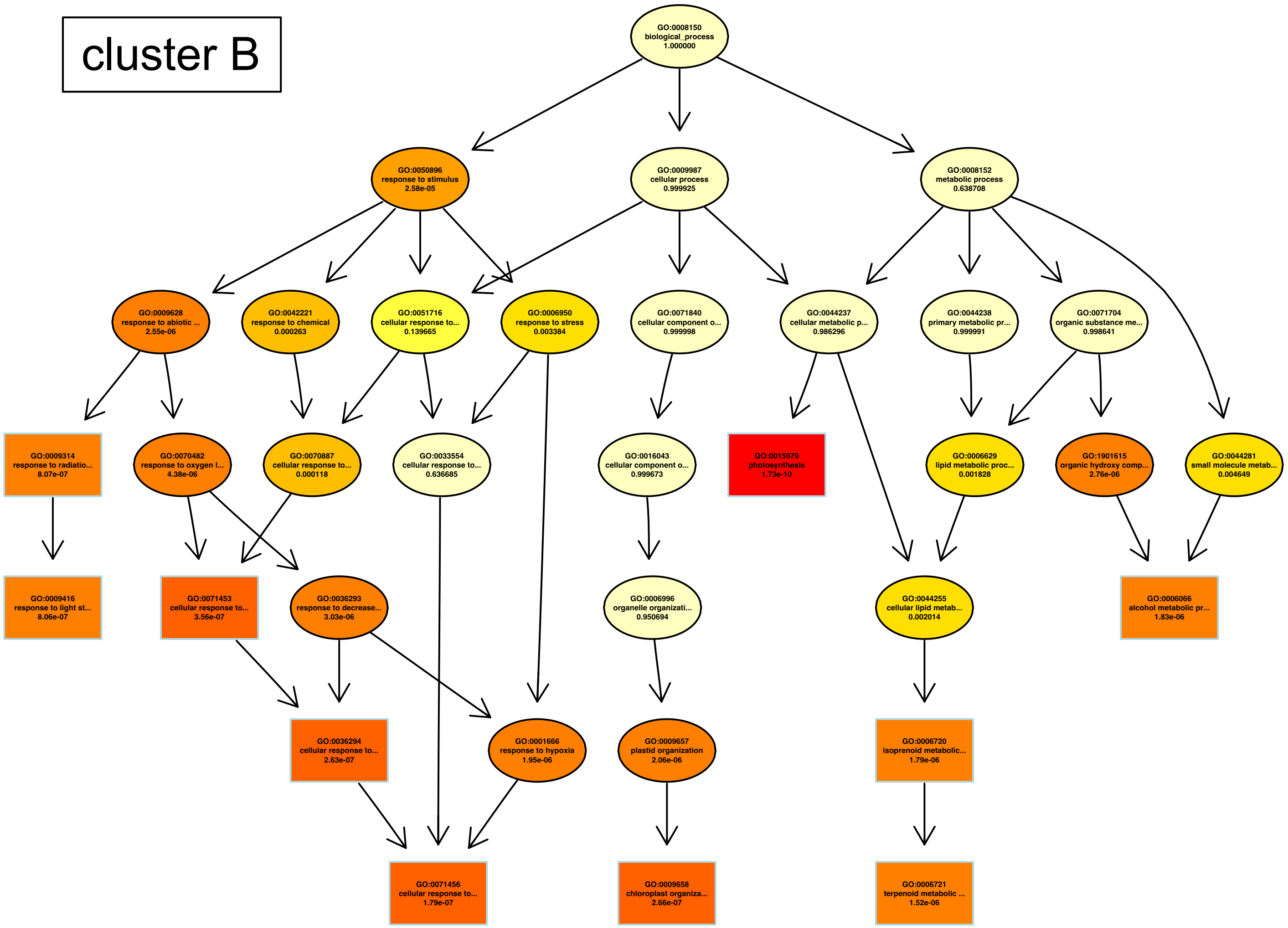

Supplementary Figure 5. High salinity suppresses the expression of genes associated with hypoxia in the aerial parts of quinoa seedlings of the lowland line, which accumulates Na^+ in the aerial parts. GO term enrichment analysis of cluster B genes (Figure 7A), which include those suppressed to a greater extent in the cotyledons of young Kd seedlings treated with high salt than in those of J075 and J100. Each enriched biological process GO term is shown in the directed acyclic graphical model. The box indicates the 10 most enriched terms out of the 50 GO terms shown in Supplementary Table 4. Different colors represent different degrees of enrichment significance, with darker colors indicating higher significance.

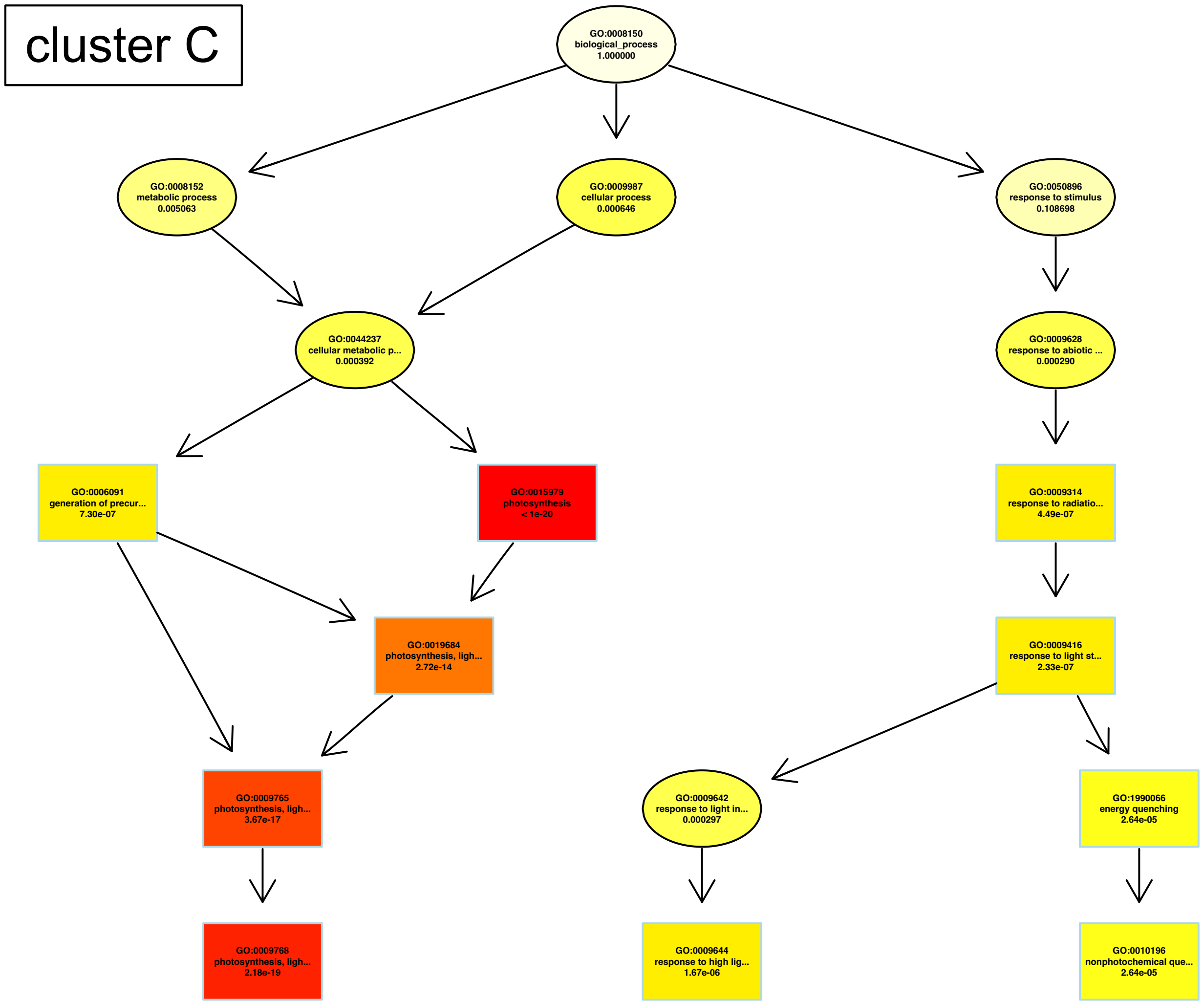

Supplementary Figure 6. High salinity suppresses the expression of photosynthesis-related genes in the aerial parts of quinoa seedlings from the lowland line, which accumulates Na^+ in the aerial parts. GO term enrichment analysis of cluster C genes (Figure 7A), which include those suppressed to a greater extent in the cotyledons of young Kd seedlings treated with high salt than in those of J075 and J100. Each enriched biological process GO term is shown in the directed acyclic graphical model. The box indicates the 10 most enriched terms out of the 50 GO terms shown in Supplementary Table 4. Different colors represent different degrees of enrichment significance, with darker colors indicating higher significance.

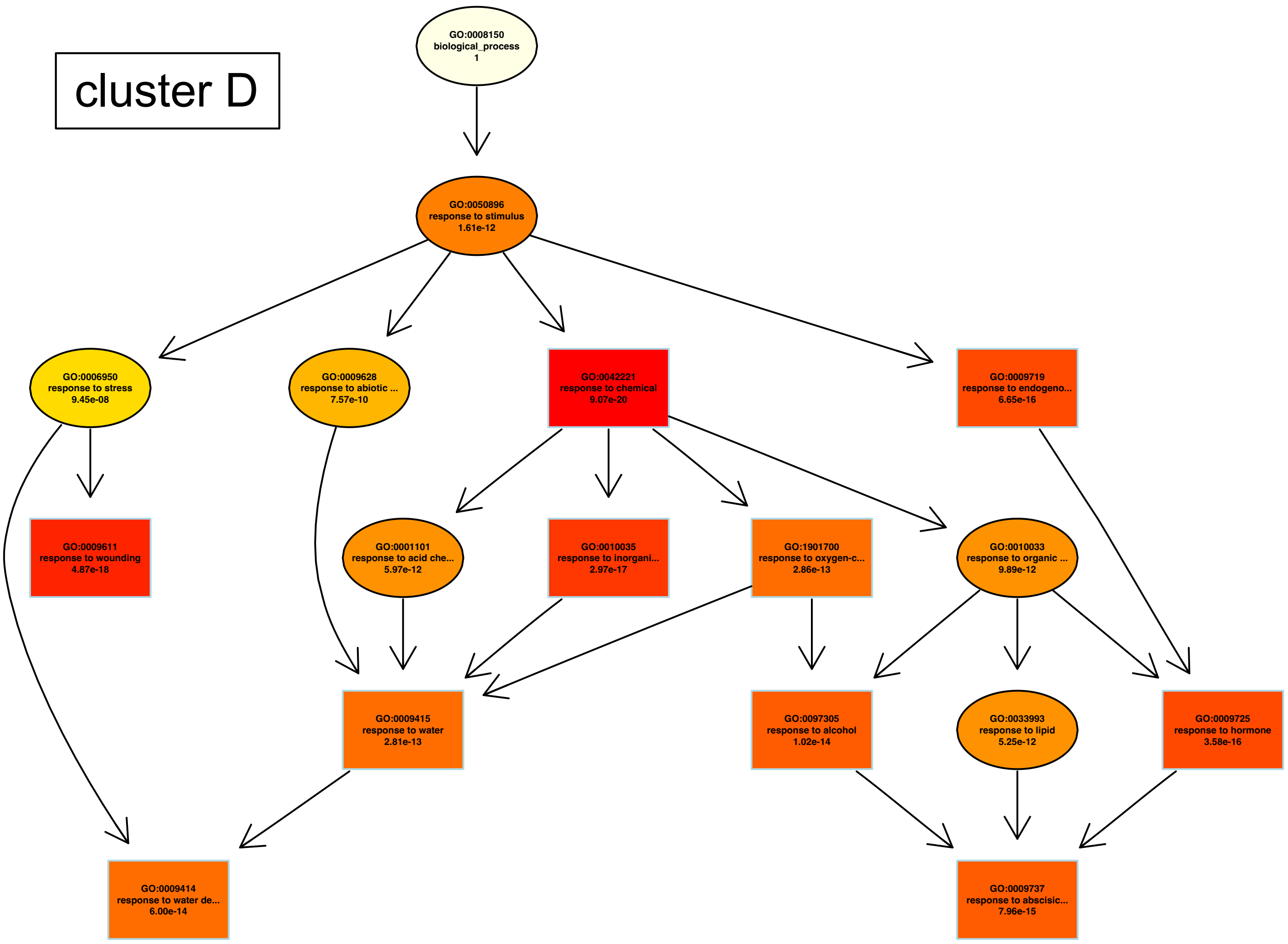

Supplementary Figure 7. High salinity induces the expression of genes associated with responses to water deprivation and ABA in the roots of quinoa seedlings from all three genotypic lines. GO term enrichment analysis of cluster D genes (Figure 7B) shows that all three genotypic lines contain more induced genes in the roots of young seedlings treated with high salt than in untreated roots. Each enriched biological process GO term is shown in the directed acyclic graphical model. The box indicates the 10 most enriched terms out of the 50 GO terms shown in Supplementary Table 5. Different colors represent different degrees of enrichment significance, with darker colors indicating higher significance.

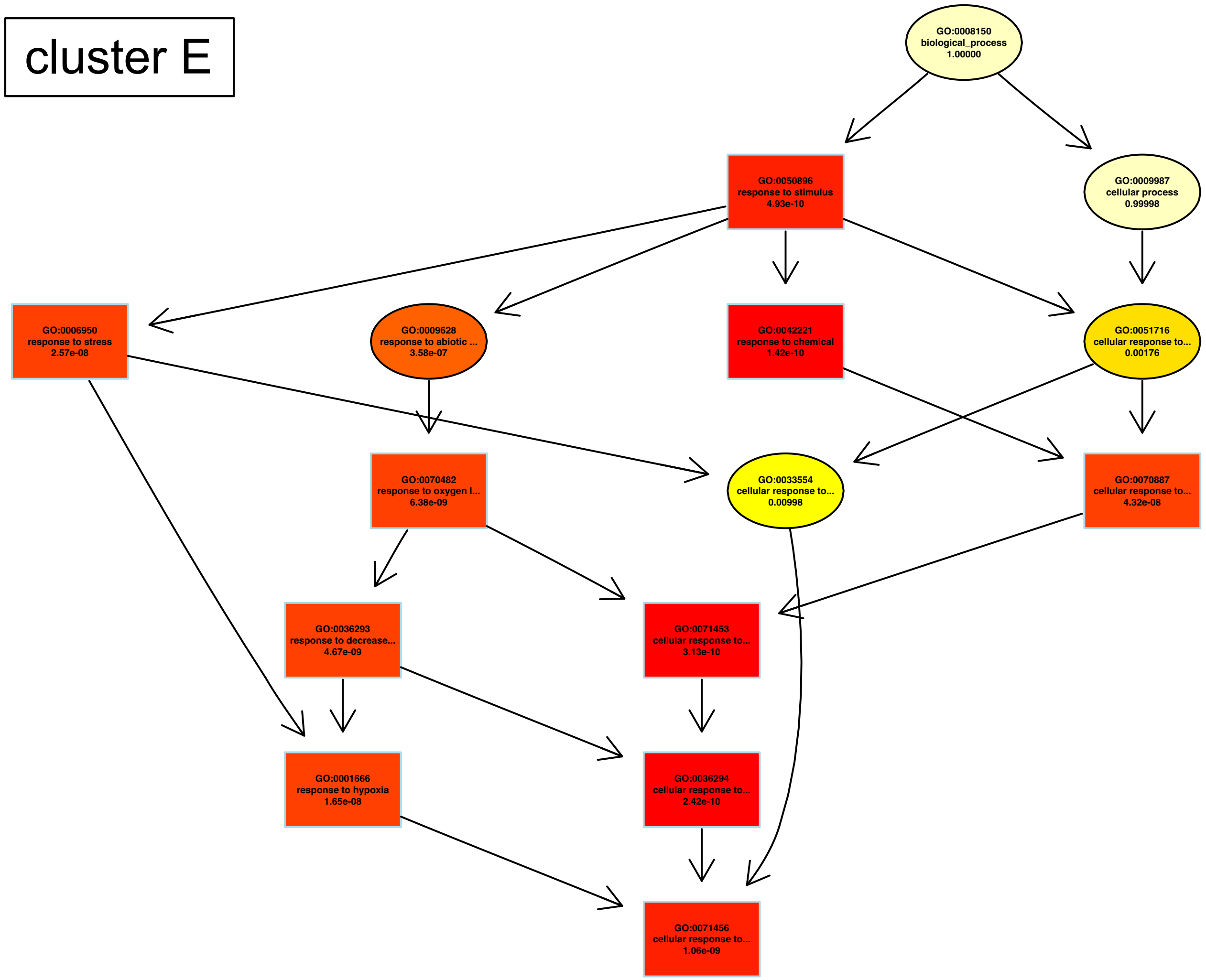

Supplementary Figure 8. High salinity greatly induces the expression of genes associated with responses to hypoxia in the roots of quinoa seedlings of the southern highland line, which does not accumulate much Na^+ in the aerial parts. GO term enrichment analysis of cluster E genes (Figure 7B), which are induced by high salt treatment to a greater extent in the roots of young J100 seedlings than in those of J075 and Kd. Each enriched biological process GO term is shown in the directed acyclic graphical model. The box indicates the 10 most enriched terms out of the 50 GO terms shown in Supplementary Table 5. Different colors represent different degrees of enrichment significance, with darker colors indicating higher significance.

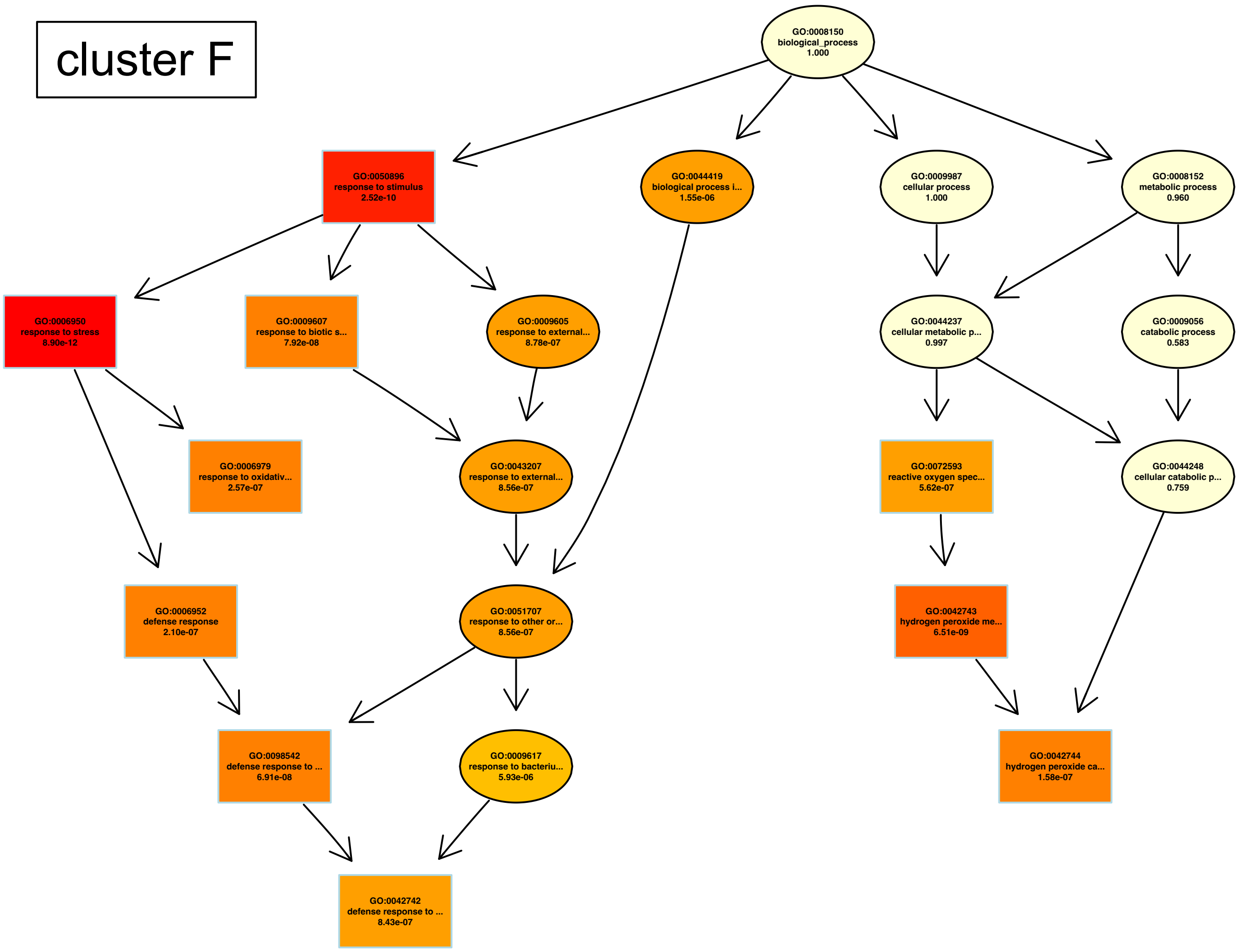

Supplementary Figure 9. High salinity induces the expression of genes associated with the defense response to bacteria and hydrogen peroxide catabolic process to a greater extent in the roots of quinoa seedlings of highland lines than in those of a lowland line. GO term enrichment analysis of cluster F genes (Figure 7B), which are induced to a greater extent in the roots of young seedlings treated with high salt in highland lines, J075 and J100, than in those of the lowland line Kd. Each enriched biological process GO term is shown in the directed acyclic graphical model. The box indicates the 10 most enriched terms out of the 50 GO terms shown in Supplementary Table 5. Different colors represent different degrees of enrichment significance, with darker colors indicating higher significance.

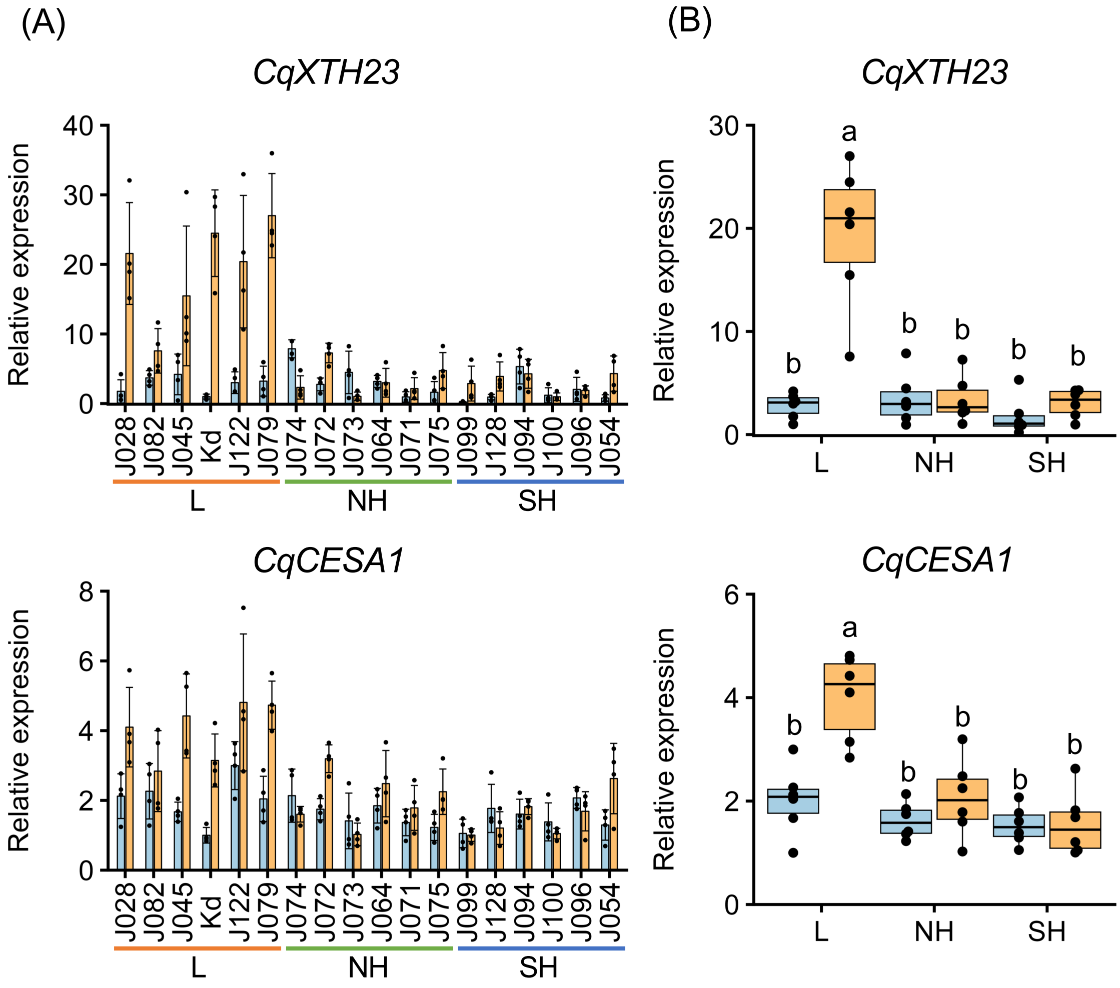

Supplementary Figure 10. Relative expression levels of cluster A DEGs in response to high salt stress in cotyledons. (A) Ten-day-old seedlings of quinoa inbred lines were treated with 0 or 600 mM NaCl for 24 h. After the treatments, gene expression in cotyledons was examined for *CqXTH23* and *CqCESA1*, which are classified into cluster A in Figure 7. L, lowland lines; NH, northern highland lines; SH, southern highland lines. To facilitate understanding of the relationship with Na^+ accumulation, the lines within each genotype are arranged from left to right in order of Na^+ content in the cotyledons, as shown in Supplementary Figure 2. The transcript levels of these genes were normalized to those of *CqUBQ10* as an internal control gene. Relative expression levels are shown as dots relative to the gene expression levels in the cotyledons in the non-salt-treated Kd seedlings. Error bars indicate SD (n = 4). (B) Average relative expression levels of *CqXTH23* and *CqCESA1* in (A) are shown as dots in the box plots. Data for lowland (L) lines include Kd, J028, J045, J079, J082, and J122; data for northern highland (NH) lines include J064, J071, J072, J073, J074, and J075; and data for southern highland (SH) lines include J054, J094, J096, J099, J100, and J128 (Supplementary Table 1).

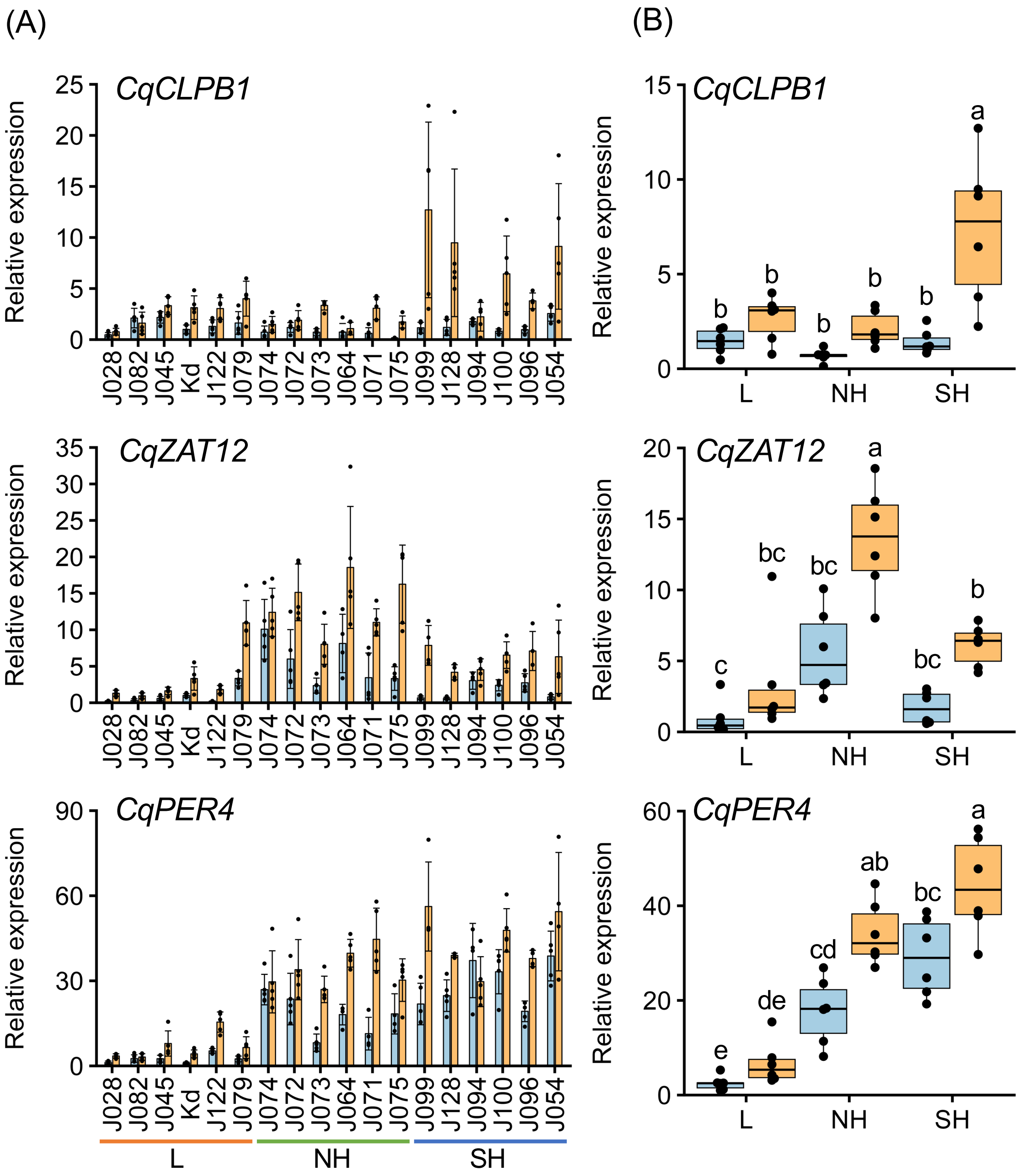

Supplementary Figure 11. Relative expression levels of cluster E and F DEGs in response to high salt stress in roots. (A) Ten-day-old seedlings of quinoa inbred lines were treated with 0 or 600 mM NaCl for 24 h. After the treatments, gene expression in roots was examined for *CqCLPB1*, *CqZAT12*, and *CqPER4*, which are classified into clusters E and F in Figure 7, respectively. L, lowland lines; NH, northern highland lines; SH, southern highland lines. To facilitate understanding of the relationship with Na^+ accumulation, the lines within each genotype are arranged from left to right in order of Na^+ content in the cotyledons, as shown in Supplementary Figure 2. The transcript levels of these genes were normalized to those of *CqUBQ10* as an internal control gene. Relative expression levels are shown as dots relative to the gene expression levels in the cotyledons in the non-salt-treated Kd seedlings. Error bars indicate SD (*n* = 4 or 5). (B) Average relative expression levels of *CqCLPB1*, *CqZAT12*, and *CqPER4* in (A) are shown as dots in the box plots. Data for lowland (L) lines include Kd, J028, J045, J079, J082, and J122; data for northern highland (NH) lines include J064, J071, J072, J073, J074, and J075; and data for southern highland (SH) lines include J054, J094, J096, J099, J100, and J128 (Supplementary Table 1).

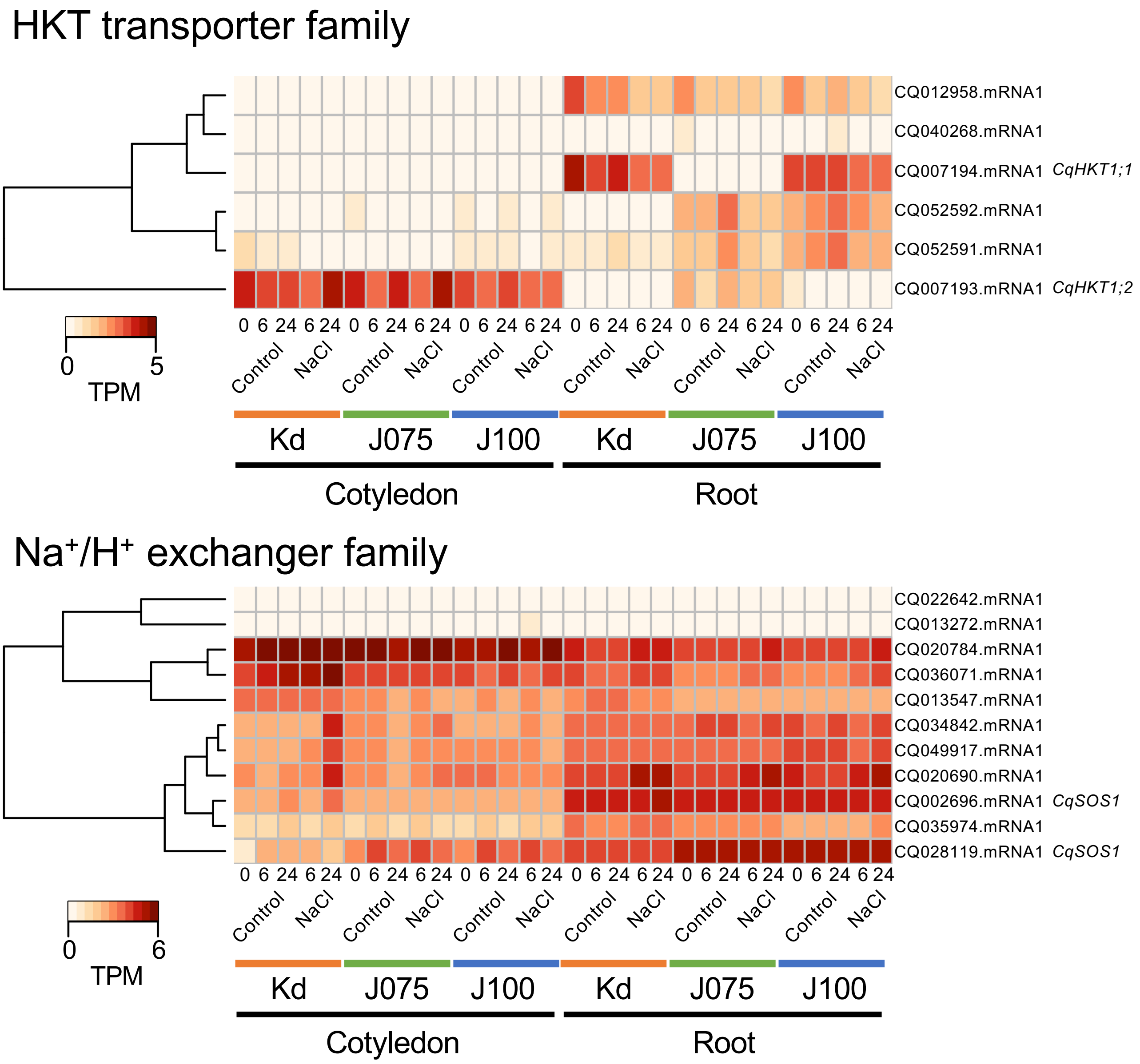

Supplementary Figure 12. Transcriptome profiling of quinoa HKT transporter and Na^+/H^+ exchanger genes in cotyledons and roots of representative quinoa inbred lines. Heatmap showing hierarchical clustering of TPM of HKT transporter and Na^+/H^+ exchanger genes in the cotyledons and roots of seedlings subjected to control conditions (0 mM NaCl) and NaCl treatment (600 mM NaCl) for 0, 6, and 24 h.

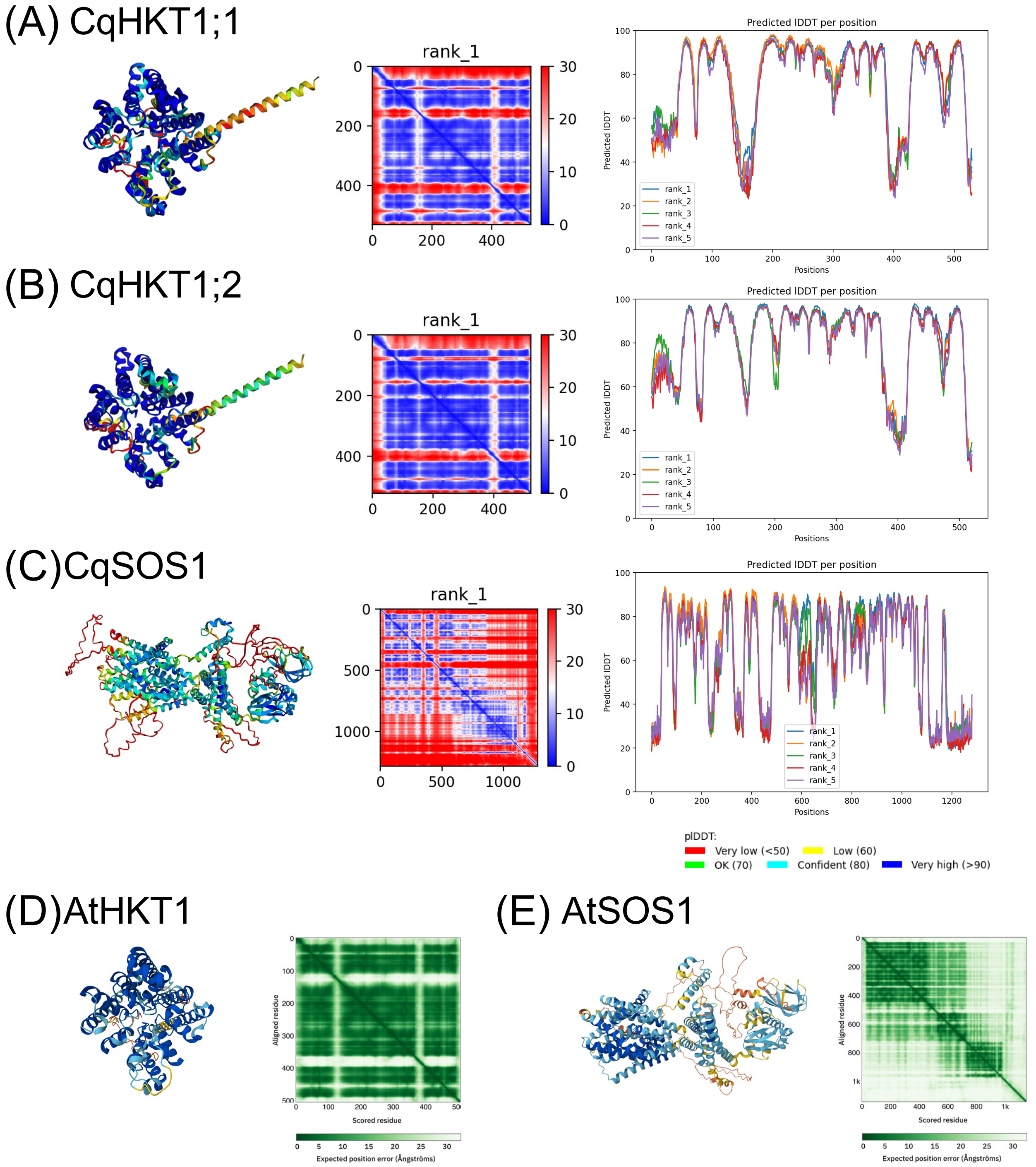

Supplementary Figure 13. 3D structures of HKT1 and SOS1 proteins from quinoa and Arabidopsis. (A-C) Alphafold predicted 3D structures (left), interactive 2D plots of predicted aligned error (PAE) (middle) and predicted local difference distance (pLDDT) values for different models (right) in CqHKT1;1, CqHKT1;2 and CqSOS1 amino acid sequences. Dark blue in the PAE plot indicates smaller errors, while red indicates larger errors. The pLDDT plot provides the best information on intra-domain confidence, with a value >90 indicating a high score. (D, E) Alphafold 3D structures with interactive 2D plots of the PAE of AtHKT1 (Q84TI7) and AtSOS1 (Q9LKW9) obtained from the AlphaFold protein structure database.

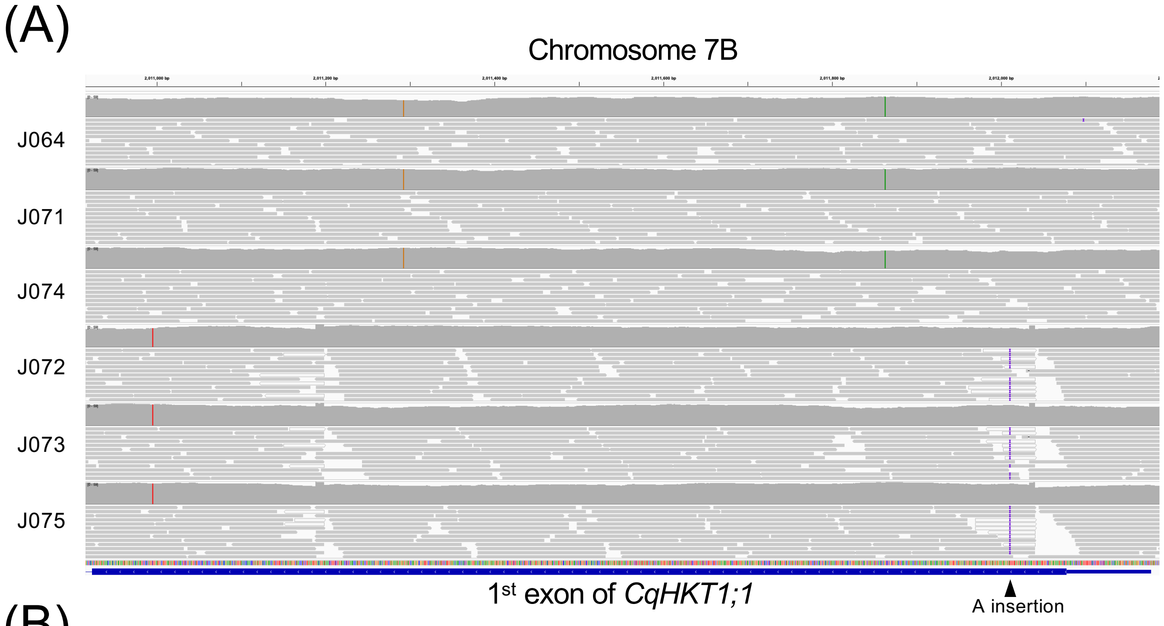

(B)

```
Reference  atg aat tac ttt tct tcc tta ggc aac aaa ttt aag caa ctt tgt ata gaa tat tat cat
            M   N   Y   F   S   S   L   G   N   K   F   K   Q   L   C   I   E   Y   Y   H
           aaa aaa ata tcc ttc att tcc cta cta tct acc atg aaa att ttc atg ttt aac tac atc
            K   K   I   S   F   I   S   L   L   S   T   M   K   I   F   M   F   N   Y   I

J072       atg aat tac ttt tct tcc tta ggc aac aaa ttt aag caa ctt tgt ata gaa tat tat cat
J073        M   N   Y   F   S   S   L   G   N   K   F   K   Q   L   C   I   E   Y   Y   H
J075       aaa aaa aat atc ctt cat ttc cct act atc tac cat gaa aat ttt cat gtt taa cta cat
            K   K   N   I   L   H   F   P   T   I   Y   H   E   N   F   H   V  Stop
```

Supplementary Figure 14. Insertion of a single adenine in the first exon in the 5′ region of the *CqHKT1;1* gene of the northern highland lines J072, J073, and J075 shifts the reading frame. (A) Visualization of a polymorphism pattern in the *CqHKT1;1* gene in northern highland lines. Read coverage (upper) and alignment (lower) for each genotype were viewed using IGV. Colored vertical lines in the reads represent single nucleotide polymorphisms (SNPs) and insertion-deletion polymorphisms (InDels) detected in the WGS data. Gene models of *CqHKT1;1* are shown in blue below the reads. Reads and their physical locations were aligned to the QQ74 reference genome (v2, id60716). (B) Two sequences at the top and bottom are genomic DNA and amino acid sequences based on the standard rules of genetic decoding of the reference line and northern highland lines (J072, J073, and J075). The insertion at position +68 in an oligo A stretch of *CqHKT1;1* in J072, J073 and J075 is marked in red. Blue letters indicate the start codon.

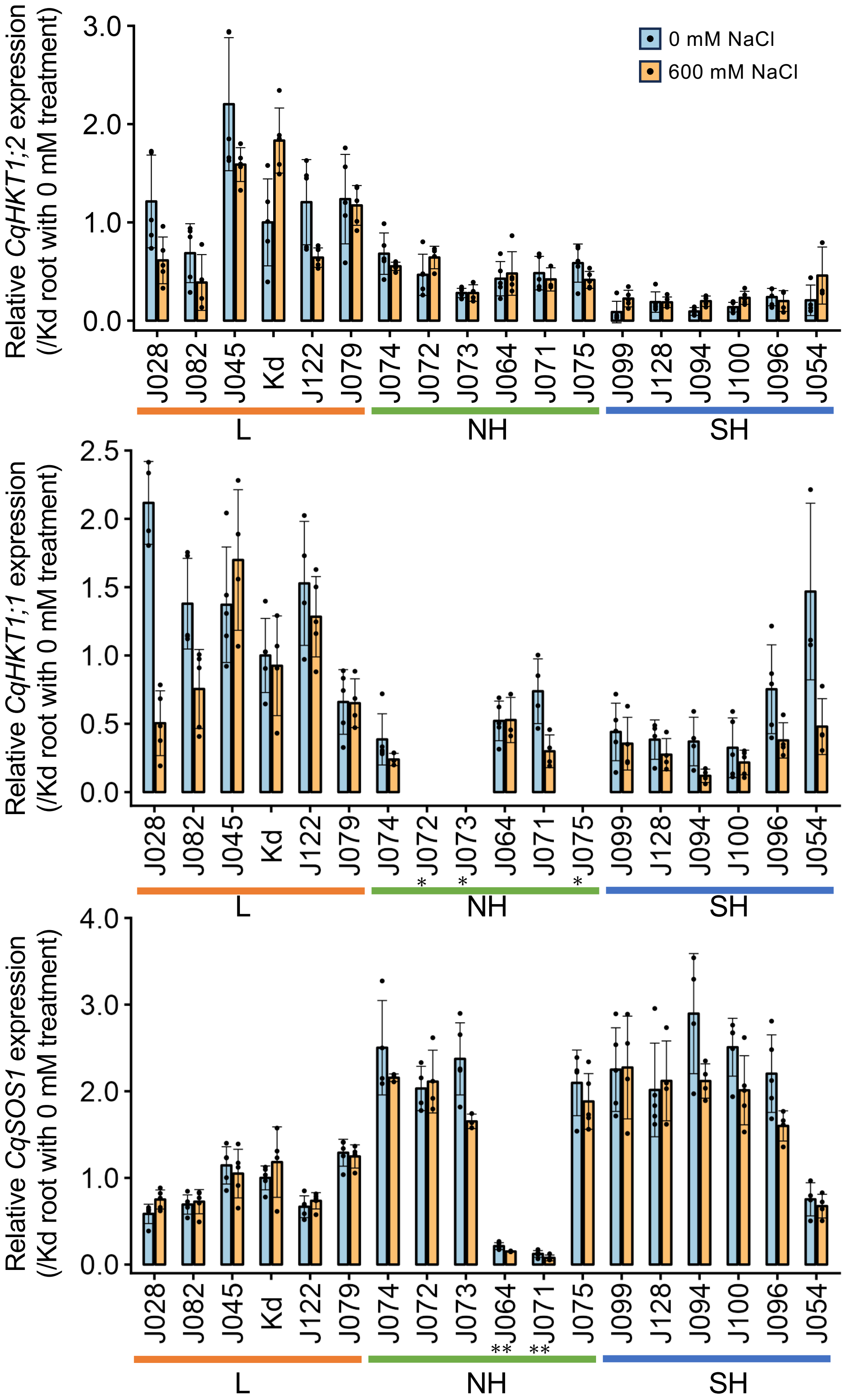

Supplementary Figure 15. Relative expression levels of Na^+ transporter genes in response to high salinity. Ten-day-old seedlings of quinoa inbred lines were treated with 0 or 600 mM NaCl for 24 h. After the treatments, gene expression in cotyledons was examined for *CqHKT1;2*, which is mainly expressed in cotyledons, and gene expression in roots was examined for *CqHKT1;1* and *CqSOS1*, which are mainly expressed in roots. L, lowland lines; NH, northern highland lines; SH, southern highland lines. To facilitate understanding of the relationship with Na^+ accumulation, the lines within each genotype are arranged from left to right in order of Na^+ content in the cotyledons, as shown in Supplementary Figure S2. The transcript levels of *CqHKT1;2*, *CqHKT1;1*, and *CqSOS1* genes were normalized to those of *CqUBQ10* as an internal control gene. Relative expression levels of *CqHKT1;1*, *CqHKT1;2*, and *CqSOS1* are shown as dots relative to the gene expression levels in each corresponding tissue in the non-salt-treated Kd seedlings. Error bars indicate SD (n = 5). Relative expression data for *CqHKT1;1* genes in J072, J073, and J075 are presented as "no gene" (*) because an insertion at position +68 in an oligoA of the gene results in a premature stop codon (Supplementary Figure 14). Relative expression data for *CqSOS1* genes in J064 and J071 are also considered "no gene" (**) due to an approximately 6.9-kb deletion in the 5′ region of the gene (Supplementary Figure 16).

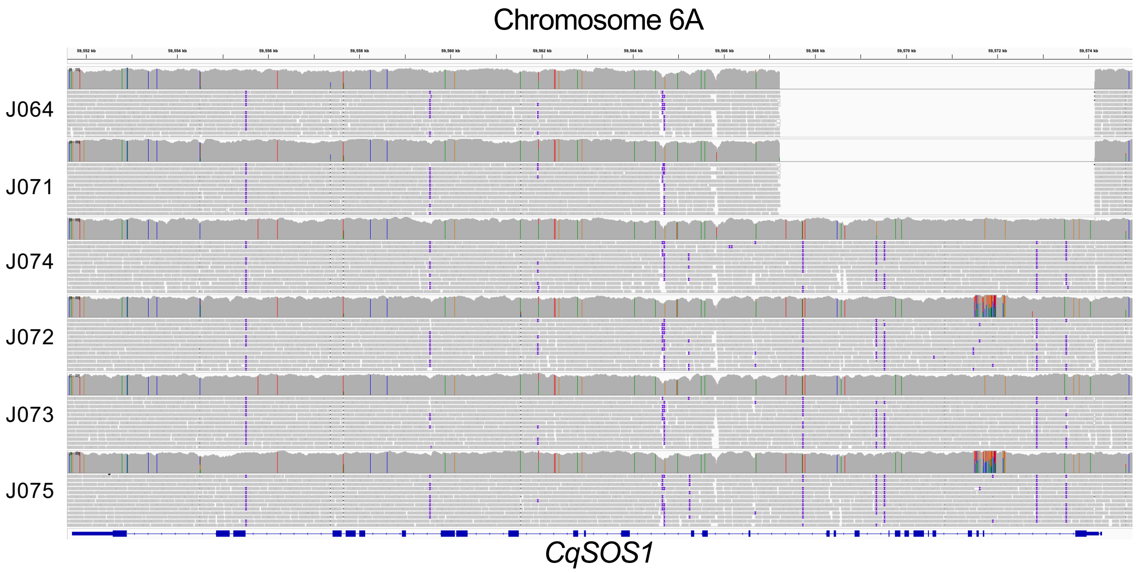

Supplementary Figure 16. Deletion of the 5′ portion of the *CqSOS1* gene in chromosome 6A of the northern highland inbred lines J064 and J071 disrupts the gene. Visualization of the polymorphism pattern in the *CqSOS1* gene in northern highland inbred lines. Read coverage (upper) and alignment (lower) for each genotype were viewed using IGV. Colored vertical lines in the reads represent SNPs and InDels detected in the WGS data. Gene models of *CqSOS1* are represented in blue below the reads. Reads and their physical locations were aligned to the QQ74 reference genome (v2, id60716).

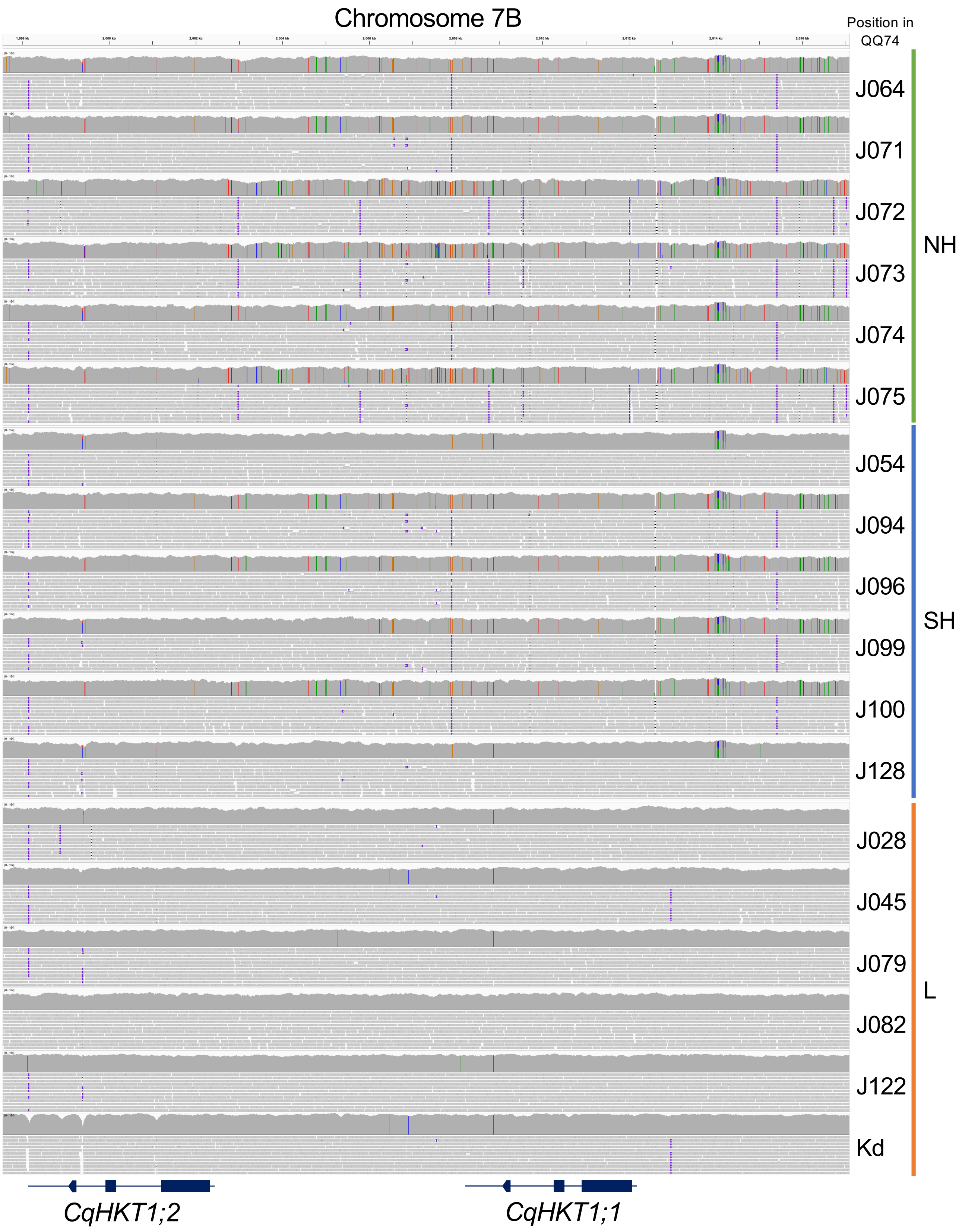

Supplementary Figure 17. Visualization of the polymorphism pattern in the upstream region of *CqHKT1* genes on chromosome 7B in selected northern highland (NH), southern highland (SH) and lowland (L) inbred lines. Read coverage (upper) and alignment (lower) for each genotype were viewed using IGV. Colored vertical lines in the reads represent SNPs and InDels detected in the WGS data. Gene models of *CqHKT1;1* and *CqHKT1;2* are shown in blue below the reads. Reads and their physical locations were aligned to the QQ74 reference genome (v2, id60716).

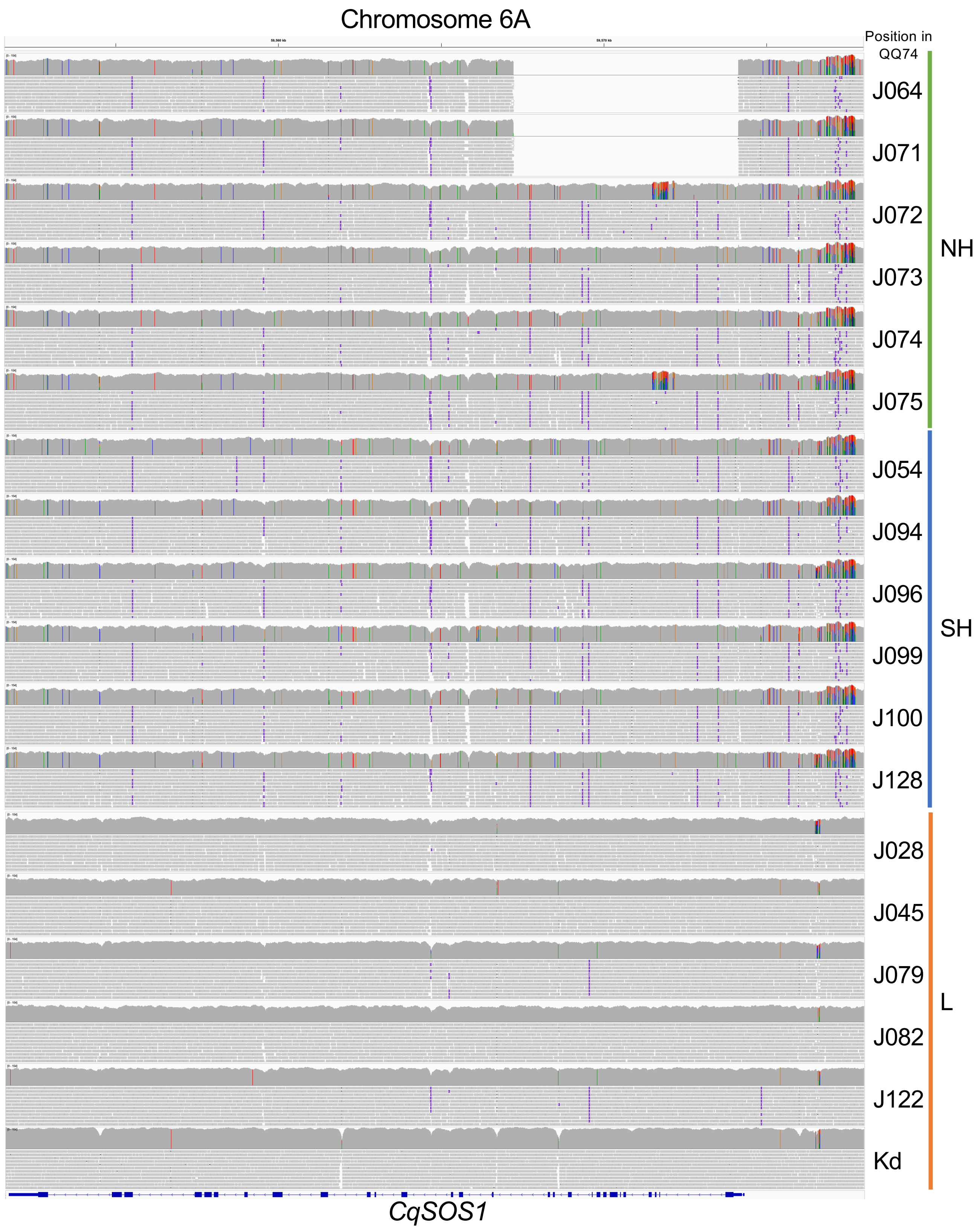

Supplementary Figure 18. Visualization of the polymorphism pattern in the upstream region of *CqSOS1* genes on chromosome 6A in selected northern highland (NH), southern highland (SH) and lowland (L) inbred lines. Read coverage (upper) and alignment (lower) for each genotype were viewed using IGV. Colored vertical lines in the reads represent SNPs and InDels detected in the WGS data. Gene models of *CqSOS1s* are shown in blue below the reads. Reads and their physical locations were aligned to the QQ74 reference genome (v2, id60716).

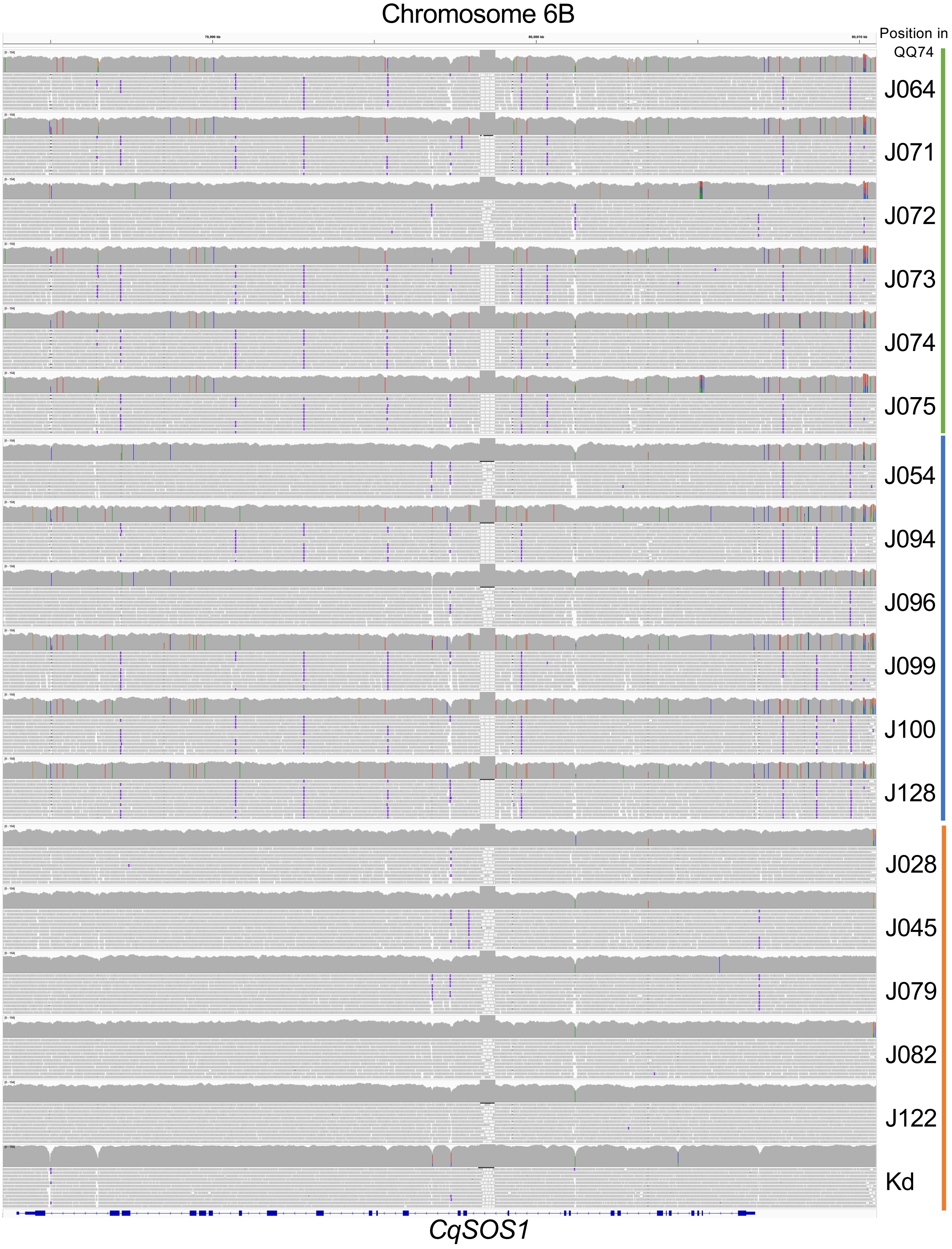

Supplementary Figure 19. Visualization of the polymorphism pattern in the upstream region of *CqSOS1* genes on chromosome 6B in selected northern highland (NH), southern highland (SH) and lowland (L) inbred lines. Read coverage (upper) and alignment (lower) for each genotype were viewed using IGV. Colored vertical lines in the reads represent SNPs and InDels detected in the WGS data. Gene models of *CqSOS1s* are shown in blue below the reads. Reads and their physical locations were aligned to the QQ74 reference genome (v2, id60716).

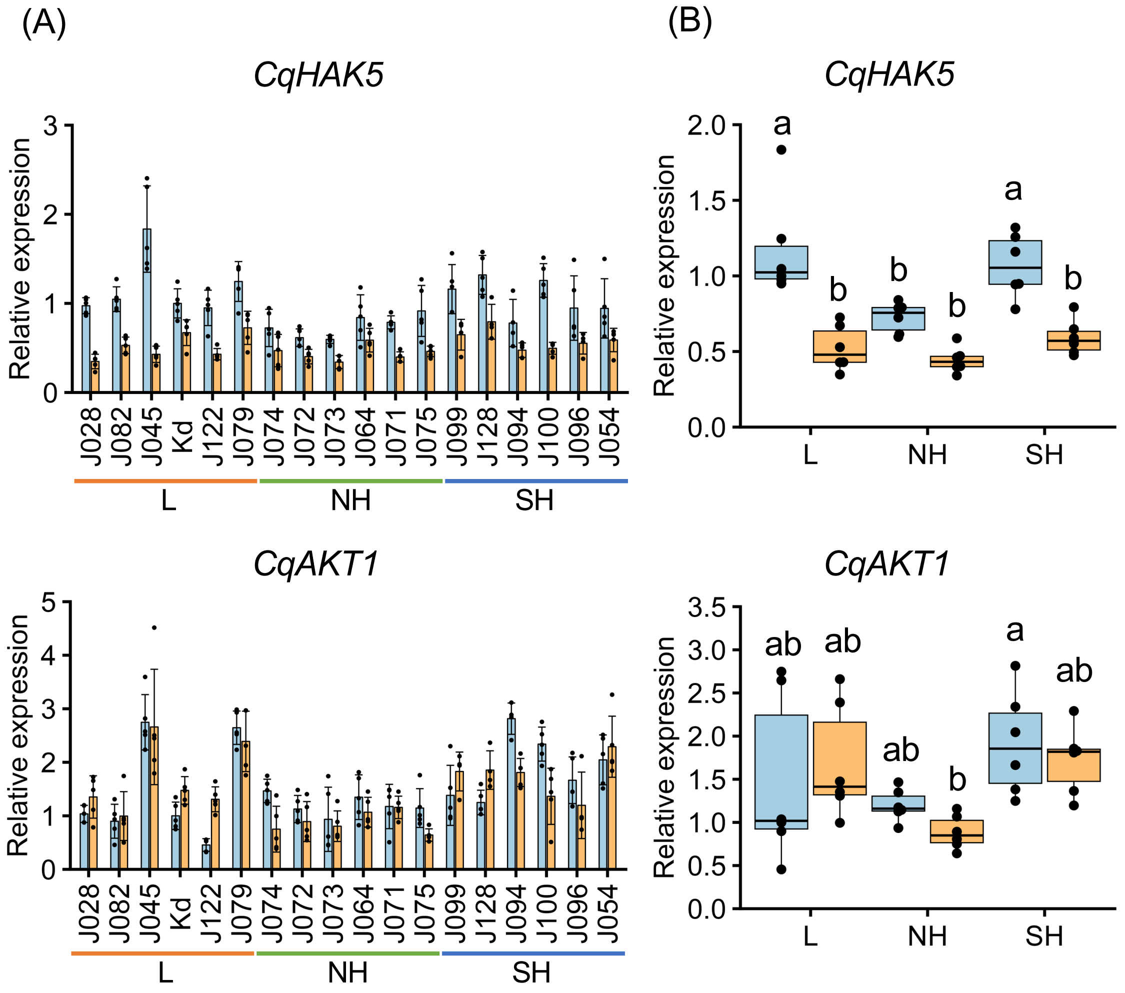

Supplementary Figure 20. Relative expression levels of K^+ transporter genes in response to high salt stress in roots. (A) Ten-day-old seedlings of quinoa inbred lines were treated with 0 or 600 mM NaCl for 24 h. After the treatments, gene expression in roots was examined for *CqAKT1* and *CqHAK5*. L, lowland lines; NH, northern highland lines; SH, southern highland lines. To facilitate understanding of the relationship with Na^+ accumulation, the lines within each genotype are arranged from left to right in order of Na^+ content in the cotyledons, as shown in Supplementary Figure 2. The transcript levels of these genes were normalized to those of *CqUBQ10* as an internal control gene. Relative expression levels are shown as dots relative to the gene expression levels in the cotyledons in the non-salt-treated Kd seedlings. Error bars indicate SD (n = 4 or 5). (B) Average relative expression levels of *CqAKT1* and *CqHAK5* in (A) are shown as dots in the box plots. Data for lowland (L) lines include Kd, J028, J045, J079, J082, and J122; data for northern highland (NH) lines include J064, J071, J072, J073, J074, and J075; and data for southern highland (SH) lines include J054, J094, J096, J099, J100, and J128 (Supplementary Table 1).

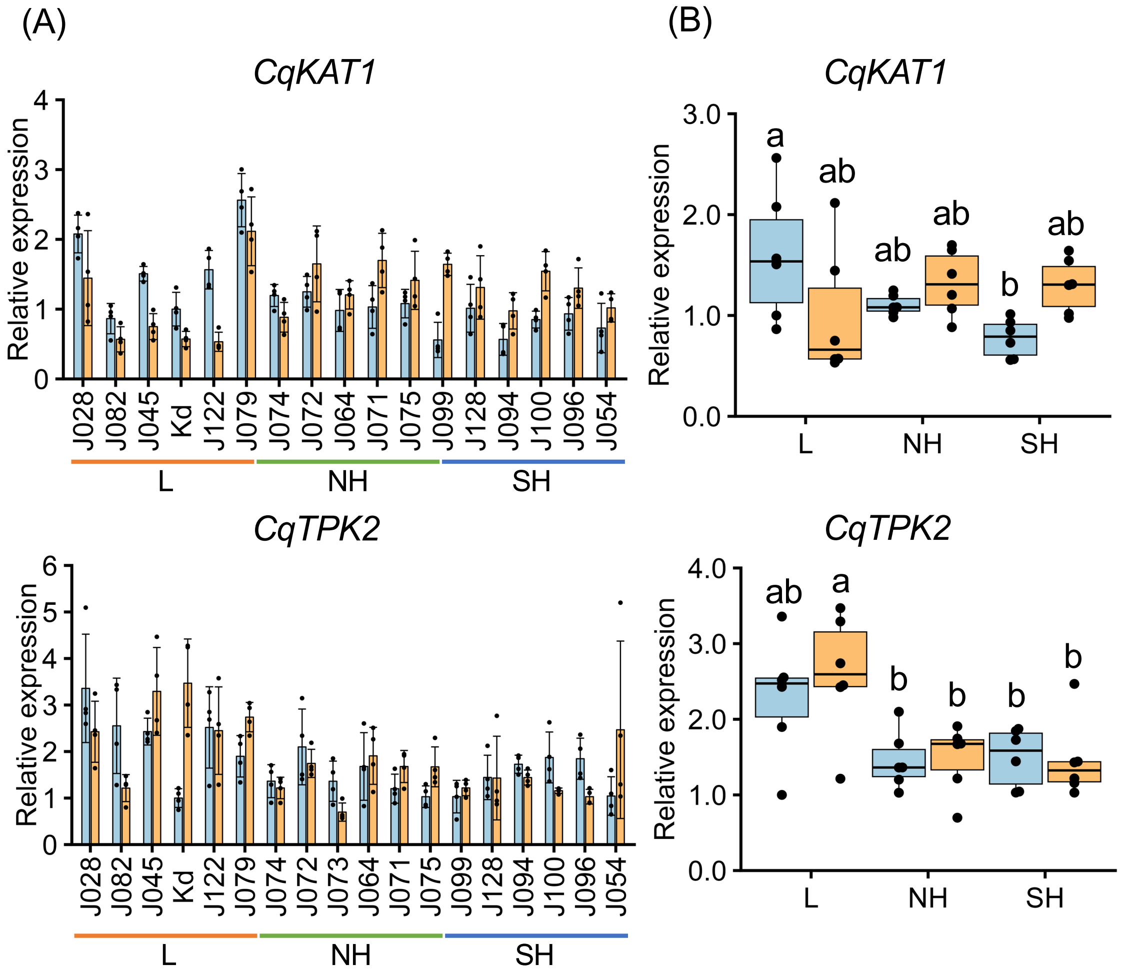

Supplementary Figure S21. Relative expression levels of K^+ transporter genes in response to high salt stress in cotyledons. (A) Ten-day-old seedlings of quinoa inbred lines were treated with 0 or 600 mM NaCl for 24 h. After the treatments, gene expression in cotyledons was examined for *CqKAT1* and *CqTPK2*. L, lowland lines; NH, northern highland lines; SH, southern highland lines. To facilitate understanding of the relationship with Na^+ accumulation, the lines within each genotype are arranged from left to right in order of Na^+ content in the cotyledons, as shown in Supplementary Figure 2. The transcript levels of these genes were normalized to those of *CqUBQ10* as an internal control gene. Relative expression levels are shown as dots relative to the gene expression levels in the cotyledons in the non-salt-treated Kd seedlings. Error bars indicate SD (n = 4). (B) Average relative expression levels of *CqKAT1* and *CqTPK2* in (A) are shown as dots in the box plots. Data for lowland (L) lines include Kd, J028, J045, J079, J082, and J122; data for northern highland (NH) lines include J064, J071, J072, J073, J074, and J075; and data for southern highland (SH) lines include J054, J094, J096, J099, J100, and J128 (Supplementary Table 1).
